# Learning the rules of peptide self-assembly through data mining with large language models

**DOI:** 10.1126/sciadv.adv1971

**Published:** 2025-03-26

**Authors:** Zhenze Yang, Sarah K. Yorke, Tuomas P. J. Knowles, Markus J. Buehler

**Affiliations:** ^1^ Laboratory for Atomistic and Molecular Mechanics, Department of Civil and Environmental Engineering, Massachusetts Institute of Technology, 77 Massachusetts Ave., Room 1-165, Cambridge, MA 02139, USA.; ^2^ Department of Materials Science and Engineering, Massachusetts Institute of Technology, 77 Massachusetts Ave., Cambridge, MA 02139, USA.; ^3^ Yusuf Hamied Department of Chemistry, University of Cambridge, Lensfield Road, Cambridge CB2 1EW, UK.; ^4^ Center for Computational Engineering, Massachusetts Institute of Technology, 77 Massachusetts Ave., Cambridge, MA 02139, USA.; ^5^ Center for Materials Science and Engineering, Massachusetts Institute of Technology, 77 Massachusetts Ave., Cambridge, MA 02139, USA.

## Abstract

Peptides are ubiquitous and important biomolecules that self-assemble into diverse structures. Although extensive research has explored the effects of chemical composition and exterior conditions on self-assembly, a systematic study consolidating these data to uncover global rules is lacking. In this work, we curate a peptide assembly database through a combination of manual processing by human experts and large language model–assisted literature mining. As a result, we collect over 1000 experimental data entries with information about peptide sequence, experimental conditions, and corresponding self-assembly phases. Using the data, machine learning models are developed, demonstrating excellent accuracy (>80%) in assembly phase classification. Moreover, we fine-tune a GPT model for peptide literature mining with the developed dataset, which markedly outperforms the pretrained model in extracting information from academic publications. This workflow can improve efficiency when exploring potential self-assembling peptide candidates, through guiding experimental work, while also deepening our understanding of the governing mechanisms.

## Teaser

An advanced LLM-based agent extracts critical insights from academic papers streamlining peptide-related research.

## INTRODUCTION

Self-assembly is a ubiquitous phenomenon in nature that plays a critical role in the formation of hierarchical biomaterials. A wide array of building blocks ranging from organic molecules and proteins to nucleic acids and amphiphilic compounds can self-assemble into nano- and microscale structures, driven by various molecular interactions (*1*–*3*). Among these building blocks, peptides present unique structural and functional characteristics with their simple yet versatile chemical compositions and noncovalent, weak intermolecular interactions. In the past few decades, a rich diversity of self-assembled peptide-based nanostructures have been reported, including tubes, fibers, ribbons, plates, and spheres (*4*–*9*). These assembled peptides serve as structured, functional biomaterials, spanning a broad spectrum of applications from drug delivery systems (*10*) and tissue engineering (*11*) to catalysis (*12*) and electronics (*13*).

The self-assembly behavior of peptides is mediated by the underlying thermodynamics and kinetics of the systems (*14*). Peptides can adopt specific organizations, such as supramolecular α helices, β sheets, and β-hairpins, which are key building blocks of specific assembled nanostructures (*15*). As a result, extensive studies have demonstrated that one can use a range of factors to manipulate a system into forming specific secondary structures. These include intrinsic parameters, such as peptide sequence (*16*) and chemical modifications (*7*), as well as extrinsic factors such as pH (*17*), temperature (*18*), solvent type (*19*–*21*), and concentration (*22*).

Now, peptide self-assembly and materials design relies largely on inherited knowledge of key motifs and interactions, known to promote self-assembly. There are two key sequences that highlight this preference for known pattern. Diphenylalanine (FF) nanofibers were produced for the first time over 20 years ago, and work since has highlighted the importance of π-π stacking interactions in short peptide assembly (*23*). Conversely, MAX1, a 20-residue β-hairpin–forming peptide, relies on its amphiphilic nature (alternating valine and lysine residues) to assemble into a rigid hydrogel (*24*). Peptides such as these examples are made through solid-phase peptide synthesis. However, this approach can be time-consuming and often requires lengthy purification before peptides can be tested experimentally. Efforts have been made to streamline the discovery of self-assembling peptides with coarse-grained (CG) molecular dynamics (MD) simulations, discussed further below (*16*). Although these simulations do help identify candidates likely to form aggregates, it is difficult to extract specific phase information, and information with respect to experimental conditions, with this approach. The simulation environment is often highly concentrated to accelerate timescales, and therefore, reproducing these results experimentally is limited (*25*).

Machine learning (ML) and artificial intelligence (AI) have emerged as unique and invaluable tools across various fields beyond computer science. For example, designing de novo proteins with target functionalities using ML and AI techniques has revolutionized bioengineering (*26*–*30*). A relevant field is polypeptide self-assembly, in which both classical ML algorithms [random forest (RF) (*31*, *32*)] and deep learning (DL) approaches [graph neural networks (*33*) or transformer-based models (*34*)] have been applied to predict the aggregation propensity of polypeptide materials given different amino acid sequences. The classical ML algorithms have shown the most promise for short peptide assembly predictions specifically thus far. A human-in-the-loop approach has been used to explore tetrapeptide hydrogelators specifically, where 160 peptides were synthesized and tested (*32*). The subsequent data were used to iteratively train an ML model that could predict peptide gelators with a success rate of 87%. Another recent work built an “AI expert” to predict short peptide sequences with aggregation potential that was found to outperform experts in the field (*31*). Although this “AI expert” does not consider assembly conditions, this work highlights the need to deviate from preconceived assembly rules that are used in the design of manual peptide materials.

It is challenging to generate large experimental datasets with synthetic molecules; thus, the datasets used for training these models are generally curated from CG MD simulations (*31*, *32*, *34*) or statistical algorithms based on physicochemical properties (*35*–*37*). Compared to experimental approaches, computational methods offer high efficiency but are constrained by their accuracy and the range of intrinsic/extrinsic parameters to which they are applicable. In addition, the aggregation propensity, a commonly predicted metric in protein science, is defined based on surface area and reflects only the capacity of proteins to aggregate, not the structural diversity of these aggregates. Therefore, collecting comprehensive experimental data is crucial for understanding the self-assembly behaviors of peptide materials and laying the groundwork for data-driven research.

When it comes to data collection from the literature, often referred to as literature mining, it becomes an intractable task for human experts to review all relevant papers due to the vast volume of publications. The recent advent of language models, such as large language models (LLMs) (*38*), introduces an innovative approach to mining academic texts written by researchers and collecting target information in a fast, automatic, and systematic manner. These LLMs, built based on graph-forming transformer architectures (*39*), have the capability to capture ultralong-distance relationships within text documents. With massive training across all types of text resources, LLMs attain the ability to comprehend the general context of human languages, making them versatile for a wide range of downstream language tasks such as graph reasoning (*40*) and text mining (*41*, *42*). Empowered by LLMs, numerous materials research studies have succeeded in extracting information about materials composition and properties from the abstract texts of academic publications (*43*–*45*). However, because of their concise, well-structured, and clear composition, abstracts can be easily processed by language models, unlike the more complex main texts with sparse and mixed-modality information (text, images, captions, references, etc.). Therefore, only a limited number of studies have undertaken literature mining of the primary texts of massive publications (*46*–*48*), and such work has used more complex processing steps to extract knowledge from papers and to then use for training LLMs. Abstracts provide only a minimal amount of information and typically lack detailed experimental procedures and results, both of which are essential for understanding and replicating self-assembly processes, thereby limiting their utility in mining for comprehensive literature data.

In this work, we curate a dataset for self-assembling short peptides with information about assembled phase and corresponding experimental conditions. This dataset is derived from publications selected from a previously established peptide self-assembly database, SAPdb (*49*), with the information being extracted by human experts. With more than 1000 data entries from the database, we are able to train and compare ML algorithms that can make accurate phase predictions based on the peptide sequence and experimental conditions. To further automate the process and increase the efficiency of literature mining, we use the manually curated dataset to fine-tune the pretrained ChatGPT model (GPT-3.5 Turbo model, referred as GPT model) via OpenAI API, which exhibits superior performance in information extraction compared to the original model, without fine-tuning. The model can be used to facilitate data collection for peptide materials, potentially providing access to previously unknown self-assembled structures while also enhancing our comprehension of the underlying principles governing their self-assembly processes.

## RESULTS

### Overall workflow

**Fig. 1.**
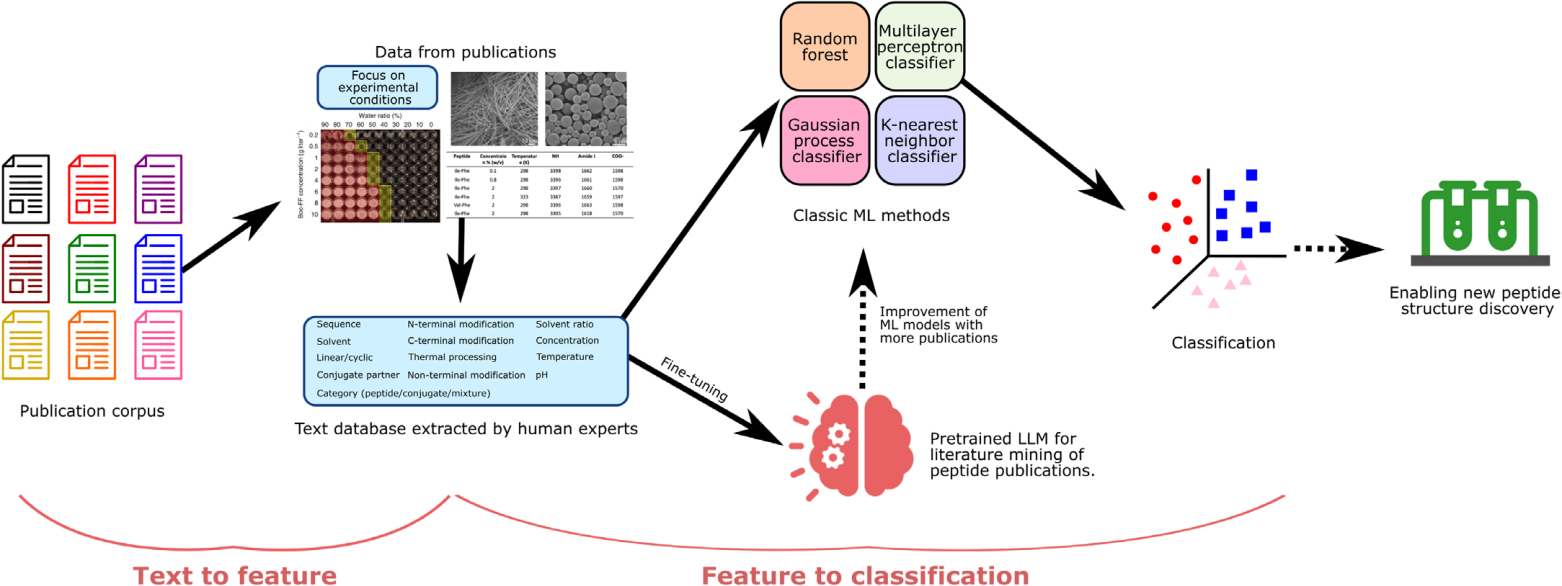
Overview of the workflow reported in this work. We first collect PDF files of publications from different journal presses and scientific databases based on the previous polypeptide database SAPdb (*49*). Here, we extract not only the peptide sequence but also experimental conditions from those previous publications and learn their impacts on the self-assembly phase of polypeptides. The selected publications are read and processed by human experts to curate the database, which is further used to train ML algorithms for predicting self-assembled structure from peptide sequences and experimental conditions. We also use the manually curated database to fine-tune an LLM to be specialized in literature mining of polypeptide publications and compare the performance with the same LLM without fine-tuning. The model can be used to extract information for new publications, reducing the time required compared to manual methods used by human experts. Moreover, by incorporating these data, we can augment our existing database, thereby further refining and enhancing our ML model for phase prediction.

**Fig. 2.**
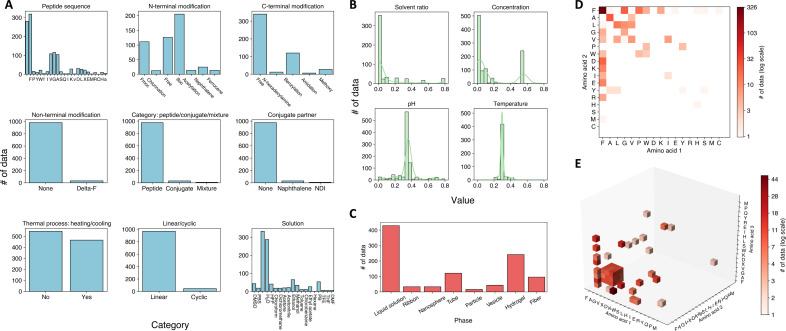
Dataset statistics. (**A**) Histogram of nine categorical features including “peptide sequence,” “N-terminal modification,” “C-terminal modification,” “Non-terminal modification,” “category: peptide/conjugate/mixture,” “conjugate partner,” “thermal process: heating/cooling,” “linear/cyclic,” and “solution” (the solution environment of the peptide). (**B**) Histogram of four numerical features including “solvent ratio” (the ratio of solvent in the solution), “concentration” (concentration of peptides), “pH” (pH of solution environment), and “temperature” (ambient temperature of experiments). All values are normalized between 0 and 1 based on min and max values. (**C**) Histogram of assembled phases. (**D**) Occurrence of dipeptide data from the academic literature. (**E**) Occurrence of tripeptide data from the academic literature.

The overall workflow of this study is depicted in Fig. 1. We first collect scientific publications related to polypeptide self-assembly from publishers directly and from scientific databases such as the PubMed database, based on a previous polypeptide database known as SAPdb (*49*). SAPdb is a collection of 1049 entries of experimentally validated short peptides (di- and tripeptides) from 301 papers. We screen the entire database based on whether each publication can be adopted into our feature template for ML predictions. In our feature template, we have nine categorical features and four numerical features as shown in Fig. 2 (A and B). Academic publications that extend experimental control beyond these specified features are not included in the database, due to their scarcity and limited utility. Following the screening process, we identified a total of 75 publications. All 75 publications are cited in Zenodo (DOI: 10.5281/zenodo.14791268). With these selected publications, we focus on extracting experimental details and the assembled phases from the main texts, which results in a total of 1012 data entries.

With the curated database, we then train ML models to predict the self-assembly phases from peptide sequence and experimental parameters. To obtain an optimal performance in the classification, we compare multiple classic ML algorithms with hyperparameter optimization, which are discussed more in detail in the following content (see Results: ML algorithms for phase prediction). In contrast to the costly and time-consuming processes of peptide synthesis and characterization, the ML model offers the ability to rapidly classify the peptides with moderate accuracy. However, the manual process of paper reading and data collection is human resource-extensive and time-consuming. Therefore, we further leverage pretrained LLM (GPT-3.5 turbo) to accelerate the process of literature mining. The LLM is engineered to perform a task known as “named entity extraction” (NER), enabling the model to efficiently extract key information from text documents based on a given targeted entity. To adapt the GPT model to be specialized in understanding scientific writing related to peptide self-assembly, the manually curated dataset is split into training and testing sets, with the training set being used to fine-tune the GPT-3.5 turbo model and testing set being implemented for performance evaluation. We demonstrate that fine-tuning can substantially enhance the performance of information extraction and requires only a small amount of data for transfer learning.

With the fine-tuned LLM assistant, we are able to perform efficient literature mining for future publications or research works that are not among selected publications such as those studying longer peptides. This future data, obtained by LLMs, can be further added into our database and used for boosting the performance of our ML model in phase classification. As a result, the proposed approach paves the way for an autonomous workflow capable of continuously collecting data from papers, augmenting the existing dataset, and refining the classification model. Furthermore, this workflow offers the potential to facilitate the design of experiments and screening of promising peptide candidates.

### Features and statistics of dataset

The statistics of curated polypeptide dataset is shown in Fig. 2. There are nine categorical features including (i) “peptide sequence,” which is the amino acid sequence of peptides and is specified for dipeptides (length of 2) or tripeptides (length of 3); (ii) “N-terminal modification,” which describes chemical modifications, such as the protecting groups, at the N terminus of peptides; (iii) “C-terminal modification,” which involves chemical modifications at the C terminus of peptides; (iv) “Non-terminal modification,” referring to chemical modifications of the R-group; (v) “category: peptide/conjugate/mixture,” indicating whether the peptide system consists of a single peptide, a conjugate, or a mixture; (vi) “conjugate partner,” identifying the conjugated peptide within a conjugate system; (vii) “thermal process: heating/cooling,” indicating the presence of temperature changes, such as heating or cooling, during the experimental process; (viii) “linear/cyclic,” distinguishing between linear and cyclic self-assembling peptides; and (9) “solution,” detailing the solution environment including information about the solvent and solute. The potential values for each categorical feature, along with their occurrence frequencies in our dataset, are visualized in Fig. 2A.

In addition to categorical features, numerical features also play a crucial role in determining the self-assembly phase of peptides. We here investigate four different numerical features as follows: (i) “solvent ratio,” which describes the volume ratio of solvent in the solution; (ii) “concentration,” defined as the concentration (mg/ml) of dissolved peptides; (iii) “pH,” which is the pH value of the solution environment; and (iv) “temperature,” denoting the ambient temperature at which the self-assembly experiment is conducted. The histograms of these numerical features are displayed in Fig. 2B. All numerical features are normalized between 0 and 1 based on the maximum and minimum values found in the database.

The combination of categorical and numerical features constitutes the complete input experimental conditions, encompassing a total of 13 features. The corresponding distribution of output self-assembly phases are shown in Fig. 2C. As shown in the figure, the most frequent phases seen in the publications is “no-assembly” case, which is not included in the original SAPdb database. Nonetheless, these instances are vital for training a ML model for phase prediction as they contribute valuable nonpositive examples to the database. Example data entries in the database are shown in table S1.

We also visualize the occurrence frequency for both dipeptides and tripeptides in our database, as depicted in Fig. 2 (D and E). For dipeptides, FF is, as expected, the most studied peptide. FF is the core recognition motif of the Alzheimer’s amyloid-β peptide and can form a wide array phases including hydrogels and hollow nanotubes under different external stimuli (*50*). For tripeptides, sequences containing FF are also commonly observed (evident in data on the *x*-*y* or *y*-*z* planes), with XFF having the highest frequency of occurrence (where X is any given amino acid). This is attributed to the wide range of experimental conditions under which XFF has been examined. Apart from phenylalanine, glycine is the second most investigated amino acids in both dipeptide and tripeptide cases. In addition, research on dipeptides predominates over tripeptides in terms of overall study volume.

### ML algorithms for phase prediction

**Fig. 3.**
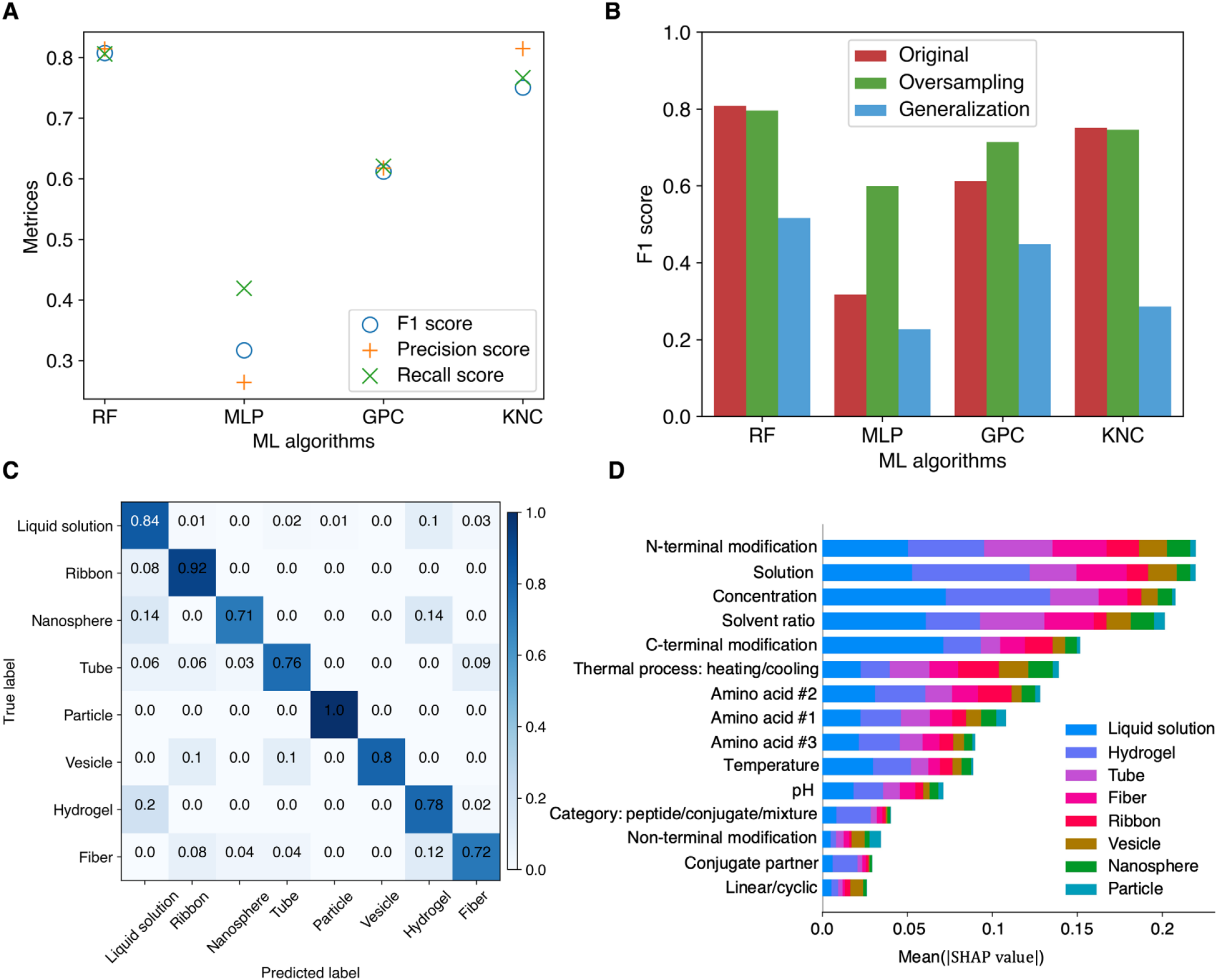
ML algorithms for phase prediction. (**A**) Performance comparison of four classical ML classifiers; RF, MLP, GPC, and KNC. *F*_1_, precision, and recall scores are used as metrics for evaluation. (**B**) Comparison of models’ performances on the imbalanced dataset (comparison between “original” and “oversampling” case) and evaluation of generalization capacity (comparison between “original” and “generalization” case). (**C**) Confusion matrix of RF model for eight different phases. (**D**) SHAP plot (*52*) for feature importance analysis.

With the curated dataset, we are now able to train ML algorithms for phase prediction with different experimental conditions and peptide sequences. Categorical features are converted to one-hot encodings based on the number of classes. All input features are then concatenated into a one-dimensional (1D) vector as input to classic ML algorithms. We compare four different classic ML algorithms: RF, multilayer perceptron (MLP) classifier, Gaussian process classifier (GPC), and K-nearest neighbor classifier (KNC). To optimize the performance of these models, we conduct a grid search of hyperparameters for each of them. More details of hyperparameter selection can be found in Materials and Methods: ML algorithms for phase classification section and the grid search results are displayed in fig. S1. Considering the dataset’s imbalance across eight distinct phases (Fig. 2C), metrics including precision, recall, and *F*_1_ scores are integrated to provide a comprehensive evaluation of four different ML algorithms. Among tested ML models, RF exhibits the best performance, achieving the highest score (precision = 0.814, recall = 0.806, and *F*_1_ = 0.808) across all three metrics (Fig. 3A). The KNC model ranks second because it exhibits a precision score comparable to that of the RF model and its recall and *F*_1_ scores are slightly lower. This indicates that KNC’s performance is more easily affected by the imbalance of the dataset. In contrast to the RF and KNC algorithms, the MLP classifier exhibits much worse accuracy, characterized by not only lower overall scores but also a large discrepancy among different metrics.

To further evaluate the performance of our ML models, we use an oversampling technique known as Synthetic Minority Oversampling Technique (SMOTE) (*51*) to address the imbalance of the dataset. The SMOTE approach generates synthetic examples of the minority class to balance the dataset by interpolating between existing minority instances. When using an oversampled dataset, the *F*_1_ scores of the MLP classifier and GPC model see a large improvement, whereas the RF and KNC algorithms maintain performances comparable to those observed with the original dataset (Fig. 3B). Among the four models evaluated, the RF model consistently demonstrates the highest accuracy when tested with a synthetic balanced dataset. However, our current method of randomly splitting the dataset may introduce bias as both the training and testing sets could contain data from the same publication. This is because a single paper often contributes multiple data entries to the dataset. To evaluate the generalization ability of our models, we partition the dataset into training and testing sets based on publications, which ensures that the data in the testing set originate from publications completely unseen by the training set. As illustrated in Fig. 3B, the *F*_1_ scores of models evaluated on the generalized test set are markedly lower than in the original scenario. However, the RF model still exhibits a relatively high *F*_1_ score (greater than 0.5), noteworthy considering that a random phase guess would only achieve a score of 0.125, given the eight phase categories in total.

On the basis of thorough evaluation of the models, we conclude that the RF model is the optimal choice for phase prediction, given its consistently superior performance across all tests. Figure 3C shows the confusion matrix predicted by the RF model, which compares the true and predicted classifications, visualizing the counts of correct and incorrect predictions across different categories. The high values observed along the diagonal of the confusion matrix underscore the model’s effectiveness and consistency in classification across different phases. All other confusion matrices for different ML algorithms tested with different situations are listed and compared in fig. S2.

After identifying an optimal model and assessing its performance, we aim to delve deeper into understanding how various input features—from peptide sequences to external stimuli—affect the final output, the self-assembly phase. To acquire interpretability of our model, we use the SHapley Additive exPlanations (SHAP) technique (*52*), which is a game theoretic approach that explains the importance of input features to the output for a ML model. Among all input features, “N-terminal modification,” “solution,” and “concentration” are the top 3 most influential features for phase classification overall (Fig. 3D). Among the two most common phases (“liquid solution” and “hydrogel”), “concentration” is the most critical feature for the “no-assembly” phase, whereas “solution” stands out as the paramount feature for the “hydrogel” phase. This is expected given that, for a new phase to be nucleated, a critical concentration must be reached. The correlations uncovered by our model offer valuable insights that can inform experimental designs. For instance, researchers can prioritize the most influential features (identified in the SHAP analysis) in their experiments when aiming to achieve a specific phase.

### LLM-assisted literature mining

**Fig. 4.**
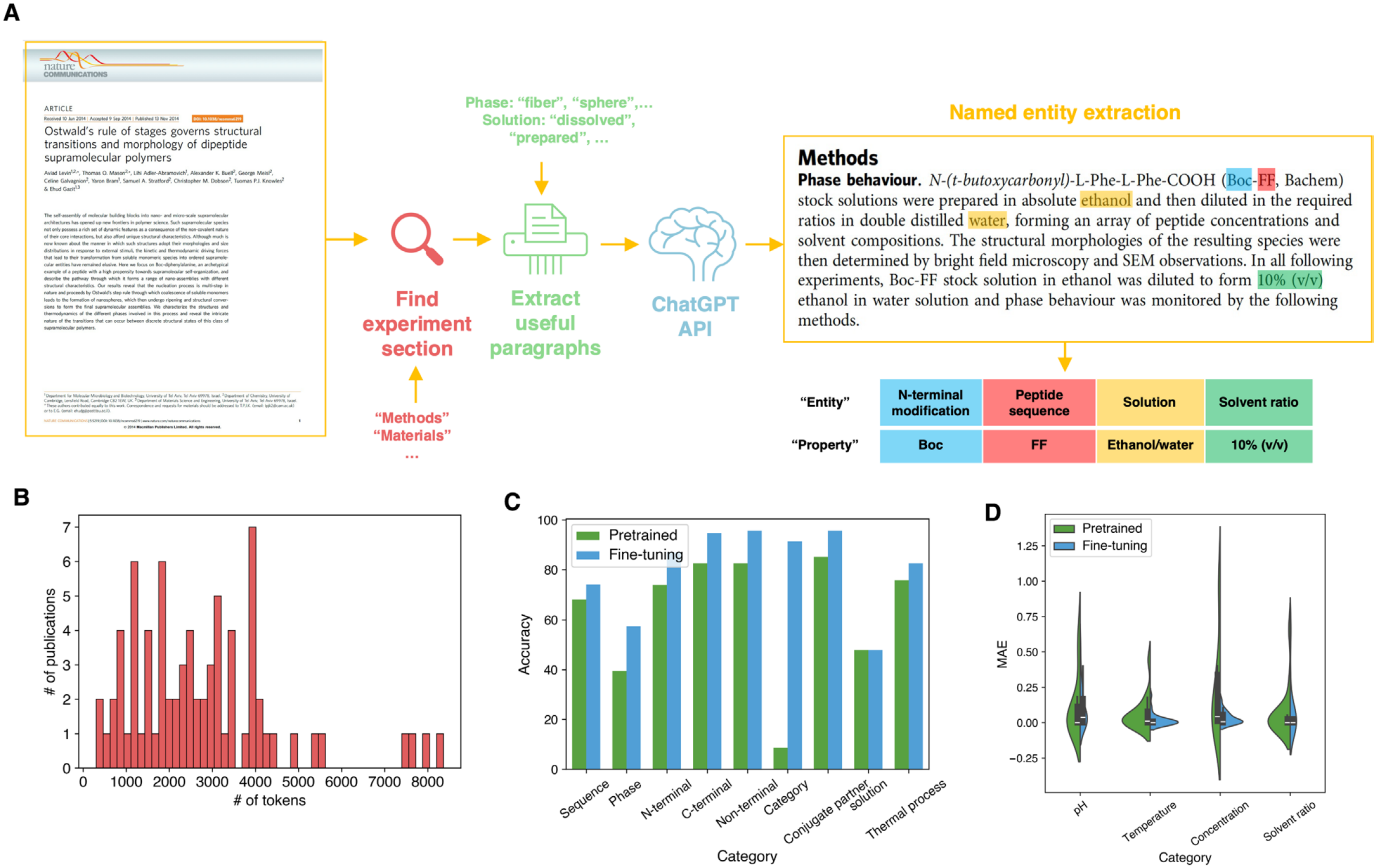
LLM-assisted literature mining. (**A**) Overall workflow of LLM-assisted literature mining: We first extract texts within the experimental section of each publication by searching for the section headings such as “Material(s),” “Method(s),” and “Experimental section(s).” Afterward, relevant paragraphs are collected based on key words related to target information. For instance, for self-assembly phase, we deliberately search for the name of phases including “fiber,” “sphere,” and others. With selected paragraphs after preprocessing, we use both pretrained and fine-tuned (with our manual dataset) to perform NER, which extracts 13 target features the from text corpus. (**B**) Histogram of length of texts after preprocessing. (**C**) Performance comparison of original pretrained GPT model and fine-tuned model for categorical feature extraction. (**D**) Performance comparison of original pretrained GPT model and fine-tuned model for numerical feature extraction.

Although the manual extraction of data from the literature by human experts offers high accuracy, it is time-consuming and labor-intensive. To streamline the information extraction process and enhance the efficiency of data collection, we here implement LLMs for automated mining of polypeptide self-assembly literature and further use our manually curated database to fine-tune and evaluate the performance of LLMs. The overall workflow of LLM-assisted literature mining is shown in Fig. 4A. We first download the PDF files of 75 academic publications from different journal publishers (fig. S3). The PDF files are then converted to texts using PDFMiner Python package (*53*). We then locate experimental sections from the whole text document by searching for section headings such as “Material(s),” “Method(s),” and “Experimental section/detail(s).” If no section heading is found, all texts are kept for further processing. Afterward, we gather relevant paragraphs based on the occurrence of key words associated with the information we aim to collect. For example, we collect paragraphs with key words such as “fibers” and “hydrogel” to obtain content related to self-assembly phases. Last, we add the abstract text, which provides a summary of each scientific paper, into the processed main text. We preprocess the texts to reduce the volume of text input, thereby meeting the token (number of words) limits for the training and inference of LLMs, and to augment the efficiency of data mining of the literature.

The distribution of token numbers for the 75 publications after preprocessing is shown as Fig. 4B. We use the OpenAI API to call the GPT model (*54*) for information extraction from these processed texts. The task is known as NER, which identifies and classifies key information elements from text into predefined categories (Fig. 4A). To enhance the model’s performance on NER for peptide literature mining, we fine-tune the GPT model with our peptide database. The peptide database is divided into training (52 papers) and testing (23 papers) sets. Given that most publications yield multiple data entries, we incorporate all data entries from the same publication into a single output, allowing the GPT model to better extract multiple sets of target entities from a single paper. We compare the performances of the original and fine-tuned GPT models by evaluating their prediction accuracy on the testing papers from the manually curated dataset.

For nine categorical features, we assess the performance of LLMs as a True-or-False task by comparing features extracted by the LLMs with those identified by human experts. The fine-tuned LLM markedly outperformed the pretrained model for all nine categorical features with an average accuracy over 80%. In comparison, the original GPT model only achieves an average accuracy of 62.7%. More specifically, fine-tuning substantially enhances the accuracy of predictions for classification queries. For example, the accuracy for identifying the feature “category: peptide/conjugate/mixture”—which determines whether the peptide system comprises a single peptide, a conjugate, or a mixture—increases from less than 10% to over 90% after fine-tuning. However, fine-tuning does not improve the model’s ability to extract information for the feature “solution.” This limitation arises from several factors: (i) “solution” often involves multiple chemicals, complicating the capture of comprehensive information; and (ii) information about the solution is frequently sparse or absent (appear in figures instead) in the processed texts used as input, making accurate extraction challenging.

In terms of the numerical features, we calculate the mean absolute error (MAE) of extracted and ground truth values to compare the accuracy for LLMs with and without fine-tuning. As clearly depicted in Fig. 4D, the fine-tuned model not only attains an MAE value but also exhibits a narrower error distribution compared to the pretrained model. This demonstrates that fine-tuning enhances both the accuracy and robustness of the GPT-3.5 turbo model for peptide literature mining. With our fine-tuned LLM, we can notably enhance the efficiency of dataset curation for learning rules of peptide self-assembly. Furthermore, this workflow can be adapted to other fields of the physical sciences as experimental conditions are crucial for scientific investigations.

### Limitations and opportunities of literature mining

Despite the notable improvements in accuracy achieved through fine-tuning our language model, it is important to recognize the existing limitations of our LLM-assisted literature mining approach, which present opportunities for further investigation and refinement in future studies. The major limitations and potential solutions to them are listed as follows (shown in fig. S4):

1) Given the limit of token numbers, we trunk the full texts of academic publications, which can eliminate important information from the text. We believe that it can be addressed by dividing the full texts into a series of chunks, which allows LLMs to process them separately rather than all at once. In addition, there is an increasingly number of open-source models with increased token limit as the field evolves.

2) In this work, we only keep the text of publications for data mining. However, figures, tables, and videos often contain essential information about self-assembly results. To overcome this limitation, multimodal LLMs hold significant promise. These advanced models are capable of simultaneously processing text, images, and videos, offering a comprehensive and integrated approach to literature mining. Furthermore, given that the graphs within the academic papers often come with captions, contrastive learning can be implemented to fine-tune these pretrained multimodal LLMs using paired text and image data.

3) We notice that, in the field of peptide self-assembly experiments, the experimental information is often distributed sparsely throughout the full text. For instance, the same group of information, such as observations from electron microscopic examination (see fig. S4), may be dispersed across various sections of a paper, including Methods, Results, and figure captions. This scattered distribution significantly complicates the task of collecting all pertinent information. To tackle the problem, during data preprocessing, we can reorganize the relevant information using regular expressions or custom parsing techniques. During the training stage, the extraction task can be enhanced with clear and task-specific prompts for sparse data collection (e.g., “Extract self-assembly phase from the Results section and temperature from the Methods section”). Last, when performing inference, chain-of-thought reasoning can be implemented to extract data in a logical and structured manner (e.g., “Extract experimental methods first and then search for corresponding results”).

4) Another challenge in mining literature of peptide self-assembly results is the frequent occurrence of multiple experiments conducted on the same peptide system to explore its morphology, composition, and properties. This results in a mixture of target information we aim to extract, involving multiple sets of experimental conditions, which confuses the LLMs we use. To address this challenge, we propose the inclusion of the experimental method as an additional entity for information extraction. In addition, prompts can be designed to specify the experiments of interest or to instruct the model to distinguish between different experiments.

## DISCUSSION

In this work, we manually collect data from the academic literature to construct a dataset of peptide self-assembly systems, with a particular emphasis on peptide sequences and experimental conditions. Using the database, classical ML algorithms, such as the RF model, are used to predict the phases of assembled nanostructures based on information regarding both internal chemical composition and external experimental stimuli. The model demonstrates high accuracy in classifying various phases and exhibits moderate generalization capabilities. By further interpreting the classification results, the importance of different experimental conditions to individual phases can be evaluated. These insights offer valuable guidance for researchers in designing experiments aimed at creating specific phases of peptide nanostructures.

We further replace the labor-intensive manual data collection process with an LLM assistant. Compared to human experts, the LLMs are capable of processing academic texts and extracting target entities in an automated and efficient manner. We used the giant pretrained GPT model and demonstrated that, by fine-tuning it with only a small set of publications, the LLM assistant can achieve impressive accuracy in extracting experimental information, significantly surpassing the performance of the original GPT model designed for general human language tasks. This fine-tuned model will facilitate the streamlining of the data collection process for future publications. The extracted data can subsequently be integrated into our database, further refining the ML algorithm trained for phase classification. Despite the notable improvements in accuracy achieved through fine-tuning our language model, it is important to recognize the existing limitations of our LLM-assisted literature mining approach, which present opportunities for further investigation and refinement in future studies. The major limitations and potential solutions to them are shown in fig. S4 and table S4.

The proposed workflow and developed models from this work can help us deepen our understanding of the self-assembly of peptide materials. However, it is important to note that the experimental data extracted from the literature tends to be biased toward specific peptides (as shown in Fig. 2, D and E) or experimental methodologies (as shown in Fig. 2, A to C). Therefore, future efforts could be directed toward incorporating data from domains that have been less explored in previous experiments. Multiple methodologies can be used to achieve this objective. For example, given the costs of conducting experiments, an active learning-based framework could be adapted to identify a minimal number of experiments required to extend knowledge beyond the current database. High-throughput screening is another potential way of conducting massive experiments in a systematic and automated manner (*55*). Compared to experiments, computational tools such as CG MD simulations are generally much faster but less accurate in phase prediction. Nevertheless, we can harness the strengths of both experimental (high-fidelity but limited in quantity) and computational (low-fidelity but abundant) techniques through the application of multifidelity learning (*56*). This approach allows the integration of both experimental and computational data to train ML models with both high accuracy and data efficiency. Current MD research and other computational studies often focus on varying peptide sequences, whereas other extrinsic factors receive less attention. In addition, because of computational costs, CG MD simulation is the only viable method to study such large peptide assemblies in a high-throughput way (*16*, *57*). However, these simulations sacrifice critical atomistic details, limiting their ability to distinguish between specific phases. Consequently, we have not included computational results in the current dataset, given the level of data volume and coverage. Looking ahead, as high-throughput MD simulations become increasingly prevalent and accessible, we anticipate acquiring a sufficiently large and comprehensive computational dataset to enhance model performance using multifidelity ML approaches in the future. Apart from physics-based experimentation and modeling, generative modeling also serves as a useful method for data generation. Model architectures from variational autoencoders and generative adversarial networks to diffusion models are widely applied to augment the data pool of materials and proteins (*58*, *59*). These approaches can be easily adapted to produce new experimental data of peptide self-assembly by learning from the existing data we have collected. Further provided with certain conditions like desired phase or constrained experimental parameters, a conditional generative model is a promising approach to assisting experimental design as we discussed in earlier sections.

Last, although this study primarily concentrates on extracting information about the self-assembly phase of peptide materials, the overarching workflow can be applied more broadly. For instance, as a structured functional material, tuning the self-assembly process is essential for accessing diverse applications. Leveraging our fine-tuned LLM for literature mining and the ML classifier for phase prediction, it becomes straightforward to gather property information about peptides from the literature and to construct a model for predicting their functions. For instance, optical (e.g., photoluminescence) and mechanical properties (e.g., modulus) are of great interest in designing peptide self-assemblies. Predicting the self-assembly phase serves as a critical foundation for exploring these functional properties. The current fine-tuned LLM can also be easily adapted to extract these properties. Given the similarity and shared language between these tasks, there is no necessity to train these models from scratch; transfer learning with a relatively small dataset suffices for precise data extraction and function predictions. Further with the generative models, we can design not only experimental parameters but also the peptide sequences to realize specific functions. These approaches will largely facilitate designing self-assembling peptide materials without conducting costly experiments.

## MATERIALS AND METHODS

### Manual processing of peptide database

We first select the data entries from the SAPdb database based on the self-assembly phase. Our database includes the top 7 most frequently occurring phases, eight inclusive of “no assembly”—hydrogel, fiber, tube, sphere, particle, ribbon, and vesicle—because other phases have too few data entries, likely making them difficult to be accurately predicted by ML algorithms. Including the nonassembly case, there are a total of eight phases. We also excluded publications that involve rare methods and techniques that cannot be incorporated within the 13 features illustrated in Fig. 2. These publications include the following cases: (i) experiments of coassembly of multiple peptides such as ref. (*60*); (ii) purely computational study such as ref. (*61*); (iii) studies on peptides that cannot split into a sequence of amino acids such as ref. (*62*); (iv) experimental control beyond solution environment, pH, and temperature such as in situ ultrasound approach (*63*), oxidation (*64*), and electromagnetic field (*65*); (v) experiments in a solution environment with more than one solvents, ref. (*66*); (vi) experiments without information of solvent/peptide concentration, ref. (*67*); and (vii) experiments with rare (less than five entries in SAPdb) solvents, additives, or chemical modifications such as ref. (*68*).

With the screening, we obtain 75 publications in total. Typically, each publication yields multiple data entries as a single study often investigates various combinations of experimental parameters. Here are the key principles we use to extract data entries from each publications: (i) For any parameters that fall within a specific range, we uniformly sample six entries within that range to constitute the data; (ii) for a given critical value of phase transition, we select two values (1.1× and 1.2×) above the critical value for one phase and two values (0.8× and 0.9×) below the critical value for the other phase; (iii) for peptide concentration, we standardize all values to the unit of mg/ml by calculating the molecular weights of peptides; (iv) for pH values, if not specified in the publications, we obtain the value based on the solvent and solute [for instance, for the pH of methanol-water mixture in ref. (*69*), the pH value is calculated to be 8.3 according to the volume ratio]; and (v) for temperature, if not specified in the publication, we presume that the experiments are performed under room temperature (25°C). By applying these rules to the publications in our database, we derive 1012 data entries.

### ML algorithms for phase classification

The input feature vector for phase classification is composed of two parts: the first from categorical features, represented through one-hot encodings, and the second from numerical features. For categorical features with multiple elements, such as “peptide sequence” and “solution,” each element is converted into a one-hot encoded vector, and these vectors are then concatenated to form a single 1D feature vector. These categorical and numerical features are combined to create an input feature vector, which has a total length of 139. The output we train the ML algorithms to predict is the self-assembly phase, which corresponds to eight different classes.

To optimize the performance of the four classical ML algorithms, grid search of hyperparameters is performed. Grid search is a method used to identify the optimal combination of hyperparameters by systematically exploring a specified search space, which is structured as a discrete grid. For each ML algorithm, we choose three independent hyperparameters to form the grid as shown in table S2. Fivefold cross-validation is implemented to compare the models under different combinations of hyperparameters. The whole curated dataset is randomly split into training (60%), validation (15%), and testing (25%) sets. All codes for hyperparameter optimization and ML calculation are written using the scikit-learn package (*70*). The results of grid search along with the optimal hyperparameter combination for four ML models are visualized in fig. S1.

In terms of the SMOTE oversampling technique, we implement it using the imbalanced-learn Python library (*71*). After oversampling, the size of the training set increases from 607 to 842 with interpolated data in minority classes. To test the generalization capacity of our ML algorithms, we split the 75 publications instead of the whole dataset into training (45 papers), validation (11 papers), and testing sets (19 papers). As a result, the final numbers of data entries for the training, validation, and testing set are 605, 151, and 256, respectively. To better interpret the impact of different features on the self-assembly phase, we implement the SHAP technique using the official SHAP Python package (*52*, *72*). Given that categorical features are represented by one-hot encodings that span multiple dimensions in the input vector, we group all dimensions related to the same feature by summing up their SHAP values.

### Text processing of the literature

We first download PDF files of 75 publications in our database and then convert the files to text. No images, tables, and videos are included after the conversion, and we exclude the supporting information/supplementary materials given the text volume limit. As we are interested in the effects of experimental conditions on the self-assembly process of peptides, we then extract experimental sections based on the section headings. These headings include “Material(s),” “Method(s),” “Material(s) and method,” “Method(s) and material(s),” “Experimental section(s),” and “Experimental detail(s).” If none of them are found in the main text, the complete text document is kept for the following processing. To determine the end of the text within the experimental sections, we search for the headings of sections that appear immediately after these experimental parts. These headings include “Result(s) and discussion(s),” “Result(s) and conclusion(s),” “Result(s),” “Discussion(s),” “Conclusion(s),” “Literature cited,” “Acknowledgement(s),” “Reference(s),” “Associated content(s),” “Author information,” and “Conflict of Interest(s).” If none of them are found, the text after the experimental section will all be included.

With the text corpus of experimental details, paragraphs with target information are then collected. The target information is searched based on a vocabulary of key words, which are relevant to the input features and output phase. The list of key words is displayed in table S3. If any of the key words are found within a paragraph, that paragraph is kept in the text for data mining; otherwise, it is removed. After collecting the relevant paragraphs, we eliminate these extremely short paragraphs (with less than 100 characters) and lines (single line with less than 9 characters), which are present mainly due to formatting errors. Upon completing these preprocessing steps, the final token count for 75 papers is presented in Fig. 4B. Considering the token limit for fine-tuning the GPT model, for processed texts that exceed this limit, we cut the beginning and ending portions to adhere to the token constraint. These texts are generally those without clear headings for different sections. This approach is taken because the beginning of a paper is the introduction section, which generally contains less information about the study itself. Similarly, the ending is often conclusions and discussions and is less critical compared to the main text.

### LLM for literature mining

In this work, we use the “GPT-3.5-turbo-0125” version of the GPT model (*54*) for both pretrained and fine-tuned cases considering the accuracy and computational costs. The maximum number of tokens that can be used for fine-tuning is 4096. Therefore, texts with more than 4096 tokens are truncated to meet the limit. We pass the name of both categorical and numerical features to our LLMs as an entity prompts to perform the NER task. The LLM is fine-tuned with batch size of 1 for only five epochs until the loss reaches a plateau. Although this study conducted fine-tuning using the OpenAI API, we observe an increasing availability of open-source LLMs, such as the Llama series. The volume of domain-specific literature in a given scientific field is considerably smaller than the training datasets used for these large models. Combined with the lower computational costs of fine-tuning methods like LoRA (*73*) compared to pretraining, this makes fine-tuning accessible to researchers with relatively modest computational resources. In addition, open-source models provide greater freedom and flexibility in prompt engineering and model customization.

Once the LLM extracts the target information from the text, we proceed to assess its accuracy manually using the following protocol: (i) For every data entry predicted by the LLM, we search for data in our manually curated dataset (considered as the ground truth) that align with the predicted peptide sequence and self-assembly phase. (ii) In cases where multiple or no direct matches are found, we evaluate the remaining target entities apart from the sequence and phase, using the ground truth data that most closely match the LLM’s predictions for accuracy calculation. (iii) For categorical features, the prediction is considered accurate only if prediction and ground truth categories fully align with each other. For features with multiple elements like “solution,” both solute and solvent need to be correctly identified for the prediction to be considered True. (iv) For numerical features, accuracy is determined by comparing the predicted and true values using the MAE. Entries lacking specific numerical values (indicated by “nan” where no values are extracted) are excluded from the MAE calculation.

## Supplementary Materials

### This PDF file includes:

Figs. S1 to S4 Tables S1 to S4 References

## References

[1] N. Stephanopoulos, J. H. Ortony, S. I. Stupp, Self-assembly for the synthesis of functional biomaterials. Acta Mater. 61, 912–930 (2013). 23457423 10.1016/j.actamat.2012.10.046 PMC3580867

[2] T. P. J. Knowles, M. J. Buehler, Nanomechanics of functional and pathological amyloid materials. Nat. Nanotechnol. 6, 469–479 (2011). 21804553 10.1038/nnano.2011.102

[3] P. J. Horn, C. L. Peterson, Chromatin higher order folding–wrapping up transcription. Science 297, 1824–1827 (2002). 12228709 10.1126/science.1074200

[4] T. Aida, E. W. Meijer, S. I. Stupp, Functional supramolecular polymers. Science 335, 813–817 (2012). 22344437 10.1126/science.1205962 PMC3291483

[5] S. Zhang, Fabrication of novel biomaterials through molecular self-assembly. Nat. Biotechnol. 21, 1171–1178 (2003). 14520402 10.1038/nbt874

[6] J. D. Hartgerink, J. R. Granja, R. A. Milligan, M. R. Ghadiri, Self-assembling peptide nanotubes. J. Am. Chem. Soc. 118, 43–50 (1996).

[7] A. Levin, T. O. Mason, L. Adler-Abramovich, A. K. Buell, G. Meisl, C. Galvagnion, Y. Bram, S. A. Stratford, C. M. Dobson, T. P. J. Knowles, E. Gazit, Ostwald’s rule of stages governs structural transitions and morphology of dipeptide supramolecular polymers. Nat. Commun. 5, 5219 (2014). 25391268 10.1038/ncomms6219

[8] K. Matsuura, K. Murasato, N. Kimizuka, Artificial peptide-nanospheres self-assembled from three-way junctions of β-sheet-forming peptides. J. Am. Chem. Soc. 127, 10148–10149 (2005). 16028908 10.1021/ja052644i

[9] H. A. Lashuel, S. R. LaBrenz, L. Woo, L. C. Serpell, J. W. Kelly, Protofilaments, filaments, ribbons, and fibrils from peptidomimetic self-assembly: Implications for amyloid fibril formation and materials science. J. Am. Chem. Soc. 122, 5262–5277 (2000). 22339465 10.1021/ja9937831

[10] M. J. Sis, M. J. Webber, Drug delivery with designed peptide assemblies. Trends Pharmacol. Sci. 40, 747–762 (2019). 31493931 10.1016/j.tips.2019.08.003

[11] S. Koutsopoulos, Self-assembling peptide nanofiber hydrogels in tissue engineering and regenerative medicine: Progress, design guidelines, and applications. J. Biomed. Mater. Res. A 104, 1002–1016 (2016). 26707893 10.1002/jbm.a.35638

[12] J. Han, H. Gong, X. Ren, X. Yan, Supramolecular nanozymes based on peptide self-assembly for biomimetic catalysis. Nano Today 41, 101295 (2021).

[13] H. A. M. Ardoña, J. D. Tovar, Peptide π-electron conjugates: Organic electronics for biology? Bioconjug. Chem. 26, 2290–2302 (2015). 26439806 10.1021/acs.bioconjchem.5b00497

[14] J. Wang, K. Liu, R. Xing, X. Yan, Peptide self-assembly: Thermodynamics and kinetics. Chem. Soc. Rev. 45, 5589–5604 (2016). 27487936 10.1039/c6cs00176a

[15] T. Li, X.-M. Lu, M.-R. Zhang, K. Hu, Z. Li, Peptide-based nanomaterials: Self-assembly, properties and applications. Bioact. Mater. 11, 268–282 (2022). 34977431 10.1016/j.bioactmat.2021.09.029 PMC8668426

[16] P. W. J. M. Frederix, R. V. Ulijn, N. T. Hunt, T. Tuttle, Virtual screening for dipeptide aggregation: Toward predictive tools for peptide self-assembly. J. Phys. Chem. Lett. 2, 2380–2384 (2011). 23795243 10.1021/jz2010573 PMC3688361

[17] Z. Li, Y. Zhu, J. B. Matson, pH-responsive self-assembling peptide-based biomaterials: Designs and applications. ACS Appl. Bio Mater. 5, 4635–4651 (2022). 10.1021/acsabm.2c00188 PMC9630172 35505454

[18] M. R. Dreher, A. J. Simnick, K. Fischer, R. J. Smith, A. Patel, M. Schmidt, A. Chilkoti, Temperature triggered self-assembly of polypeptides into multivalent spherical micelles. J. Am. Chem. Soc. 130, 687–694 (2008). 18085778 10.1021/ja0764862 PMC2855373

[19] A. Nandakumar, Y. Ito, M. Ueda, Solvent effects on the self-assembly of an amphiphilic polypeptide incorporating α-helical hydrophobic blocks. J. Am. Chem. Soc. 142, 20994–21003 (2020). 33272014 10.1021/jacs.0c03425

[20] M. J. Krysmann, V. Castelletto, J. E. McKendrick, L. A. Clifton, I. W. Hamley, P. J. F. Harris, S. M. King, Self-assembly of peptide nanotubes in an organic solvent. Langmuir 24, 8158–8162 (2008). 18572891 10.1021/la800942n

[21] T. O. Mason, D. Y. Chirgadze, A. Levin, L. Adler-Abramovich, E. Gazit, T. P. J. Knowles, A. K. Buell, Expanding the solvent chemical space for self-assembly of dipeptide nanostructures. ACS Nano 8, 1243–1253 (2014). 24422499 10.1021/nn404237f

[22] C. J. Bowerman, B. L. Nilsson, A reductive trigger for peptide self-assembly and hydrogelation. J. Am. Chem. Soc. 132, 9526–9527 (2010). 20405940 10.1021/ja1025535

[23] M. Reches, E. Gazit, Casting metal nanowires within discrete self-assembled peptide nanotubes. Science 300, 625–627 (2003). 12714741 10.1126/science.1082387

[24] J. P. Schneider, D. J. Pochan, B. Ozbas, K. Rajagopal, L. Pakstis, J. Kretsinger, Responsive hydrogels from the intramolecular folding and self-assembly of a designed peptide. J. Am. Chem. Soc. 124, 15030–15037 (2002). 12475347 10.1021/ja027993g

[25] A. van Teijlingen, T. Tuttle, Beyond tripeptides two-step active machine learning for very large data sets. J. Chem. Theory Comput. 17, 3221–3232 (2021). 33904712 10.1021/acs.jctc.1c00159 PMC8278388

[26] J. L. Watson, D. Juergens, N. R. Bennett, B. L. Trippe, J. Yim, H. E. Eisenach, W. Ahern, A. J. Borst, R. J. Ragotte, L. F. Milles, B. I. M. Wicky, N. Hanikel, S. J. Pellock, A. Courbet, W. Sheffler, J. Wang, P. Venkatesh, I. Sappington, S. V. Torres, A. Lauko, V. De Bortoli, E. Mathieu, S. Ovchinnikov, R. Barzilay, T. S. Jaakkola, F. D. Maio, M. Baek, D. Baker, De novo design of protein structure and function with RFdiffusion. Nature 620, 1089–1100 (2023). 37433327 10.1038/s41586-023-06415-8 PMC10468394

[27] B. Ni, D. L. Kaplan, M. J. Buehler, Generative design of de novo proteins based on secondary-structure constraints using an attention-based diffusion model. Chem 9, 1828–1849 (2023). 37614363 10.1016/j.chempr.2023.03.020 PMC10443900

[28] Z. Yang, Y.-C. Hsu, M. J. Buehler, Generative multiscale analysis of de novo proteome-inspired molecular structures and nanomechanical optimization using a VoxelPerceiver transformer model. J. Mech. Phys. Solids 170, 105098 (2023).

[29] W. Lu, D. L. Kaplan, M. J. Buehler, Generative modeling, design, and analysis of spider silk protein sequences for enhanced mechanical properties. Adv. Funct. Mater. 34, 2311324 (2024).

[30] A. Ghafarollahi, M. J. Buehler, ProtAgents: Protein discovery *via* large language model multi-agent collaborations combining physics and machine learning. Digit. Discov. 3, 1389–1409 (2024). 38993729 10.1039/d4dd00013g PMC11235180

[31] R. Batra, T. D. Loeffler, H. Chan, S. Srinivasan, H. Cui, I. V. Korendovych, V. Nanda, L. C. Palmer, L. A. Solomon, H. C. Fry, S. K. R. S. Sankaranarayanan, Machine learning overcomes human bias in the discovery of self-assembling peptides. Nat. Chem. 14, 1427–1435 (2022). 36316409 10.1038/s41557-022-01055-3 PMC9844539

[32] T. Xu, J. Wang, S. Zhao, D. Chen, H. Zhang, Y. Fang, N. Kong, Z. Zhou, W. Li, H. Wang, Accelerating the prediction and discovery of peptide hydrogels with human-in-the-loop. Nat. Commun. 14, 3880 (2023). 37391398 10.1038/s41467-023-39648-2 PMC10313671

[33] S. Kang, M. Kim, J. Sun, M. Lee, K. Min, Prediction of protein aggregation propensity via data-driven approaches. ACS Biomater Sci. Eng. 9, 6451–6463 (2023). 37844262 10.1021/acsbiomaterials.3c01001

[34] J. Wang, Z. Liu, S. Zhao, T. Xu, H. Wang, S. Z. Li, W. Li, Deep learning empowers the discovery of self-assembling peptides with over 10 trillion sequences. Adv. Sci. 10, 2301544 (2023). 10.1002/advs.202301544 PMC10625107 37749875

[35] A.-M. Fernandez-Escamilla, F. Rousseau, J. Schymkowitz, L. Serrano, Prediction of sequence-dependent and mutational effects on the aggregation of peptides and proteins. Nat. Biotechnol. 22, 1302–1306 (2004). 15361882 10.1038/nbt1012

[36] R. Zambrano, M. Jamroz, A. Szczasiuk, J. Pujols, S. Kmiecik, S. Ventura, AGGRESCAN3D (A3D): Server for prediction of aggregation properties of protein structures. Nucleic Acids Res. 43, W306–W313 (2015). 25883144 10.1093/nar/gkv359 PMC4489226

[37] K. Sankar, S. R. Krystek Jr., S. M. Carl, T. Day, J. K. X. Maier, AggScore: Prediction of aggregation-prone regions in proteins based on the distribution of surface patches. Proteins 86, 1147–1156 (2018). 30168197 10.1002/prot.25594

[38] W. X. Zhao, K. Zhou, J. Li, T. Tang, X. Wang, Y. Hou, Y. Min, B. Zhang, J. Zhang, Z. Dong, Y. Du, C. Yang, Y. Chen, Z. Chen, J. Jiang, R. Ren, Y. Li, X. Tang, Z. Liu, P. Liu, J.-Y. Nie, J.-R. Wen, Survey of Large Language Models. arXiv:2303.18223 [cs.CL] (2023).

[39] A. Vaswani, N. Shazeer, N. Parmar, J. Uszkoreit, L. Jones, A. N. Gomez, Ł. Kaiser, I. Polosukhin, Attention is all you need, in *Proceedings of the 31st International Conference on Neural Information Processing Systems* (Curran Associates Inc., 2017), pp. 6000–6010.

[40] M. J. Buehler, Accelerating Scientific Discovery with Generative Knowledge Extraction, Graph-Based Representation, and Multimodal Intelligent Graph Reasoning. arXiv:2403.119961 [cs.LG] (2024).

[41] T. B. Brown, B. Mann, N. Ryder, M. Subbiah, J. Kaplan, P. Dhariwal, A. Neelakantan, P. Shyam, G. Sastry, A. Askell, S. Agarwal, A. Herbert-Voss, G. Krueger, T. Henighan, R. Child, A. Ramesh, D. M. Ziegler, J. Wu, C. Winter, C. Hesse, M. Chen, E. Sigler, M. Litwin, S. Gray, B. Chess, J. Clark, C. Berner, S. McCandlish, A. Radford, I. Sutskever, D. Amodei, Language Models are Few-Shot Learners. arXiv:2005.14165 [cs.CL] (2020).

[42] H. Touvron, L. Martin, K. Stone, P. Albert, A. Almahairi, Y. Babaei, N. Bashlykov, S. Batra, P. Bhargava, S. Bhosale, D. Bikel, L. Blecher, C. C. Ferrer, M. Chen, G. Cucurull, D. Esiobu, J. Fernandes, J. Fu, W. Fu, B. Fuller, C. Gao, V. Goswami, N. Goyal, A. Hartshorn, S. Hosseini, R. Hou, H. Inan, M. Kardas, V. Kerkez, M. Khabsa, I. Kloumann, A. Korenev, P. S. Koura, M.-A. Lachaux, T. Lavril, J. Lee, D. Liskovich, Y. Lu, Y. Mao, X. Martinet, T. Mihaylov, P. Mishra, I. Molybog, Y. Nie, A. Poulton, J. Reizenstein, R. Rungta, K. Saladi, A. Schelten, R. Silva, E. M. Smith, R. Subramanian, X. E. Tan, B. Tang, R. Taylor, A. Williams, J. X. Kuan, P. Xu, Z. Yan, I. Zarov, Y. Zhang, A. Fan, M. Kambadur, S. Narang, A. Rodriguez, R. Stojnic, S. Edunov, T. Scialom, Llama 2: Open Foundation and Fine-Tuned Chat Models. arXiv:2307.09288 [cs.CL] (2023).

[43] V. Tshitoyan, J. Dagdelen, L. Weston, A. Dunn, Z. Rong, O. Kononova, K. A. Persson, G. Ceder, A. Jain, Unsupervised word embeddings capture latent knowledge from materials science literature. Nature 571, 95–98 (2019). 31270483 10.1038/s41586-019-1335-8

[44] T. Gupta, M. Zaki, N. M. A. Krishnan, Mausam, MatSciBERT: A materials domain language model for text mining and information extraction. NPJ Comput. Mater. 8, 102 (2022).

[45] L. Foppiano, G. Lambard, T. Amagasa, M. Ishii, Mining experimental data from Materials Science literature with Large Language Models: an evaluation study. arXiv:2401.11052 [cs.CL] (2024).

[46] R. K. Luu, M. J. Buehler, BioinspiredLLM: Conversational large language model for the mechanics of biological and bio-inspired materials. Adv. Sci. 11, 2306724 (2024). 10.1002/advs.202306724 PMC10933662 38145334

[47] M. J. Buehler, MechGPT, a language-based strategy for mechanics and materials modeling that connects knowledge across scales, disciplines, and modalities. Appl. Mech. Rev. 76, 21001 (2024).

[48] M. J. Buehler, Cephalo: Multi-Modal Vision-Language Models for Bio-Inspired Materials Analysis and Design. arXiv:2405.19076 [cs.CV] (2024).

[49] D. Mathur, H. Kaur, A. Dhall, N. Sharma, G. P. S. Raghava, SAPdb: A database of short peptides and the corresponding nanostructures formed by self-assembly. Comput. Biol. Med. 133, 104391 (2021). 33892308 10.1016/j.compbiomed.2021.104391

[50] P. Tamamis, L. Adler-Abramovich, M. Reches, K. Marshall, P. Sikorski, L. Serpell, E. Gazit, G. Archontis, Self-assembly of phenylalanine oligopeptides: insights from experiments and simulations. Biophys. J. 96, 5020–5029 (2009). 19527662 10.1016/j.bpj.2009.03.026 PMC2712050

[51] N. V. Chawla, K. W. Bowyer, L. O. Hall, W. P. Kegelmeyer, SMOTE: Synthetic minority over-sampling technique. J. Artif. Int. Res. 16, 321–357 (2002).

[52] S. M. Lundberg, S.-I. Lee, A unified approach to interpreting model predictions, in *Advances in Neural Information Processing Systems 30*, I. Guyon, U. V. Luxburg, S. Bengio, H. Wallach, R. Fergus, S. Vishwanathan, R. Garnett, Eds. (Curran Associates Inc., 2017), pp. 4765–4774.

[53] pdfminer developers, pdfminer.six PDF parser and analyzer (2024).

[54] OpenAI, GPT-3.5-turbo-0125 (2023).

[55] S. Marchesan, Y. Qu, L. J. Waddington, C. D. Easton, V. Glattauer, T. J. Lithgow, K. M. Mc Lean, J. S. Forsythe, P. G. Hartley, Self-assembly of ciprofloxacin and a tripeptide into an antimicrobial nanostructured hydrogel. Biomaterials 34, 3678–3687 (2013). 23422591 10.1016/j.biomaterials.2013.01.096

[56] C. Fare, P. Fenner, M. Benatan, A. Varsi, E. O. Pyzer-Knapp, A multi-fidelity machine learning approach to high throughput materials screening. NPJ Comput. Mater. 8, 257 (2022).

[57] P. W. J. M. Frederix, G. G. Scott, Y. M. Abul-Haija, D. Kalafatovic, C. G. Pappas, N. Javid, N. T. Hunt, R. V. Ulijn, T. Tuttle, Exploring the sequence space for (tri-)peptide self-assembly to design and discover new hydrogels. Nat. Chem. 7, 30–37 (2015). 25515887 10.1038/nchem.2122

[58] B. Sanchez-Lengeling, A. Aspuru-Guzik, Inverse molecular design using machine learning: Generative models for matter engineering. Science 361, 360–365 (2018). 30049875 10.1126/science.aat2663

[59] Y.-C. Hsu, Z. Yang, M. J. Buehler, Generative design, manufacturing, and molecular modeling of 3D architected materials based on natural language input. APL Mater. 10, 41107 (2022).

[60] R. Xing, S. Li, N. Zhang, G. Shen, H. Möhwald, X. Yan, Self-assembled injectable peptide hydrogels capable of triggering antitumor immune response. Biomacromolecules 18, 3514–3523 (2017). 28721731 10.1021/acs.biomac.7b00787

[61] C. Guo, Y. Luo, R. Zhou, G. Wei, Triphenylalanine peptides self-assemble into nanospheres and nanorods that are different from the nanovesicles and nanotubes formed by diphenylalanine peptides. Nanoscale 6, 2800–2811 (2014). 24468750 10.1039/c3nr02505e

[62] I. Bhardwaj, D. Jha, P. Admane, A. K. Panda, V. Haridas, Self-assembling tryptophan-based designer peptides as intracellular delivery vehicles. Bioorg. Med. Chem. Lett. 26, 672–676 (2016). 26631316 10.1016/j.bmcl.2015.11.041

[63] C. G. Pappas, P. W. J. M. Frederix, T. Mutasa, S. Fleming, Y. M. Abul-Haija, S. M. Kelly, A. Gachagan, D. Kalafatovic, J. Trevino, R. V. Ulijn, S. Bai, Alignment of nanostructured tripeptide gels by directional ultrasonication. Chem. Commun. 51, 8465–8468 (2015). 10.1039/c5cc02049b 25891849

[64] R. P. Lyon, W. M. Atkins, Self-assembly and gelation of oxidized glutathione in organic solvents. J. Am. Chem. Soc. 123, 4408–4413 (2001). 11457225 10.1021/ja0040417

[65] G. Baskar, M. Ravi, J. J. Panda, A. Khatri, B. Dev, R. Santosham, S. Sathiya, C. S. Babu, V. S. Chauhan, S. K. Rayala, G. Venkatraman, Efficacy of dipeptide-coated magnetic nanoparticles in lung cancer models under pulsed electromagnetic field. Cancer Invest. 35, 431–442 (2017). 28537455 10.1080/07357907.2017.1318894

[66] L. Adler-Abramovich, E. Gazit, Controlled patterning of peptide nanotubes and nanospheres using inkjet printing technology. J. Pept. Sci. 14, 217–223 (2008). 18035858 10.1002/psc.963

[67] C. A. E. Hauser, R. Deng, A. Mishra, Y. Loo, U. Khoe, F. Zhuang, D. W. Cheong, A. Accardo, M. B. Sullivan, C. Riekel, J. Y. Ying, U. A. Hauser, Natural tri- to hexapeptides self-assemble in water to amyloid β-type fiber aggregates by unexpected α-helical intermediate structures. Proc. Natl. Acad. Sci. U.S.A. 108, 1361–1366 (2011). 21205900 10.1073/pnas.1014796108 PMC3029732

[68] T. Liebmann, S. Rydholm, V. Akpe, H. Brismar, Self-assembling Fmoc dipeptide hydrogel for in situ 3D cell culturing. BMC Biotechnol. 7, 88 (2007). 18070345 10.1186/1472-6750-7-88 PMC2235856

[69] S. Yadav, V. Rai, M. Mahato, M. Singh, R. S. Deka, K. A. Sharma, Vitamin E–TPGS stabilized self-assembled tripeptide nanostructures for drug delivery. Curr. Top. Med. Chem. 15, 1227–1235 (2015). 25858135 10.2174/1568026615666150330111348

[70] F. Pedregosa, G. Varoquaux, A. Gramfort, B. Michel, V. Thirion, O. Grisel, M. Blondel, R. Prettenhofer, P. Weiss, V. Dubourg, J. Vanderplas, A. Passos, D. Cournapeau, M. Brucher, M. Perrot, E. Duchesnay, Scikit-learn: Machine learning in Python. J. Mach. Learn. Res. 12, 2825–2830 (2011).

[71] G. Lemaître, F. Nogueira, C. K. Aridas, Imbalanced-learn: A python toolbox to tackle the curse of imbalanced datasets in machine learning. J. Mach. Learn. Res. 18, 1–5 (2017).

[72] S. M. Lundberg, G. Erion, H. Chen, A. DeGrave, J. M. Prutkin, B. Nair, R. Katz, J. Himmelfarb, N. Bansal, S.-I. Lee, From local explanations to global understanding with explainable AI for trees. Nat. Mach. Intell. 2, 2522–5839 (2020). 10.1038/s42256-019-0138-9 PMC7326367 32607472

[73] E. J. Hu, Y. Shen, P. Wallis, Z. Allen-Zhu, Y. Li, S. Wang, L. Wang, W. Chen, LoRA: Low-Rank Adaptation of Large Language Models, in *International Conference on Learning Representations (ICLR)* (ICLR, 2022).

[74] N. S. de Groot, T. Parella, F. X. Aviles, S. Ventura, Ile-Phe dipeptide self-assembly: Clues to amyloid formation. Biophys. J. 92, 1732–1741 (2007). 17172307 10.1529/biophysj.106.096677 PMC1796831

[75] M. Cao, C. Cao, L. Zhang, D. Xia, H. Xu, Tuning of peptide assembly through force balance adjustment. J. Colloid Interface Sci. 407, 287–295 (2013). 23871602 10.1016/j.jcis.2013.06.051

