# Supplementary material for: Learning the rules of peptide self-assembly through data mining with large language models
Source: Sci Adv. 2025 Mar 26;11(13):eadv1971. doi: 10.1126/sciadv.adv1971 (PMC11939049; doi:10.1126/sciadv.adv1971)
Supplement: Supplementary file 1 — Figs. S1 to S4 Tables S1 to S4 References [file sciadv.adv1971_sm.pdf]

Science Advances
AAAS

Supplementary Materials for

Learning the rules of peptide self-assembly through data mining with large language models

Zhenze Yang *et al.*

Corresponding author: Markus J. Buehler, mbuehler@mit.edu; Tuomas P. J. Knowles, tpjk2@cam.ac.uk

Sci. Adv. **11**, eadv1971 (2025)
DOI: 10.1126/sciadv.adv1971

This PDF file includes:

Figs. S1 to S4
Tables S1 to S4
References

Supplementary figures and tables

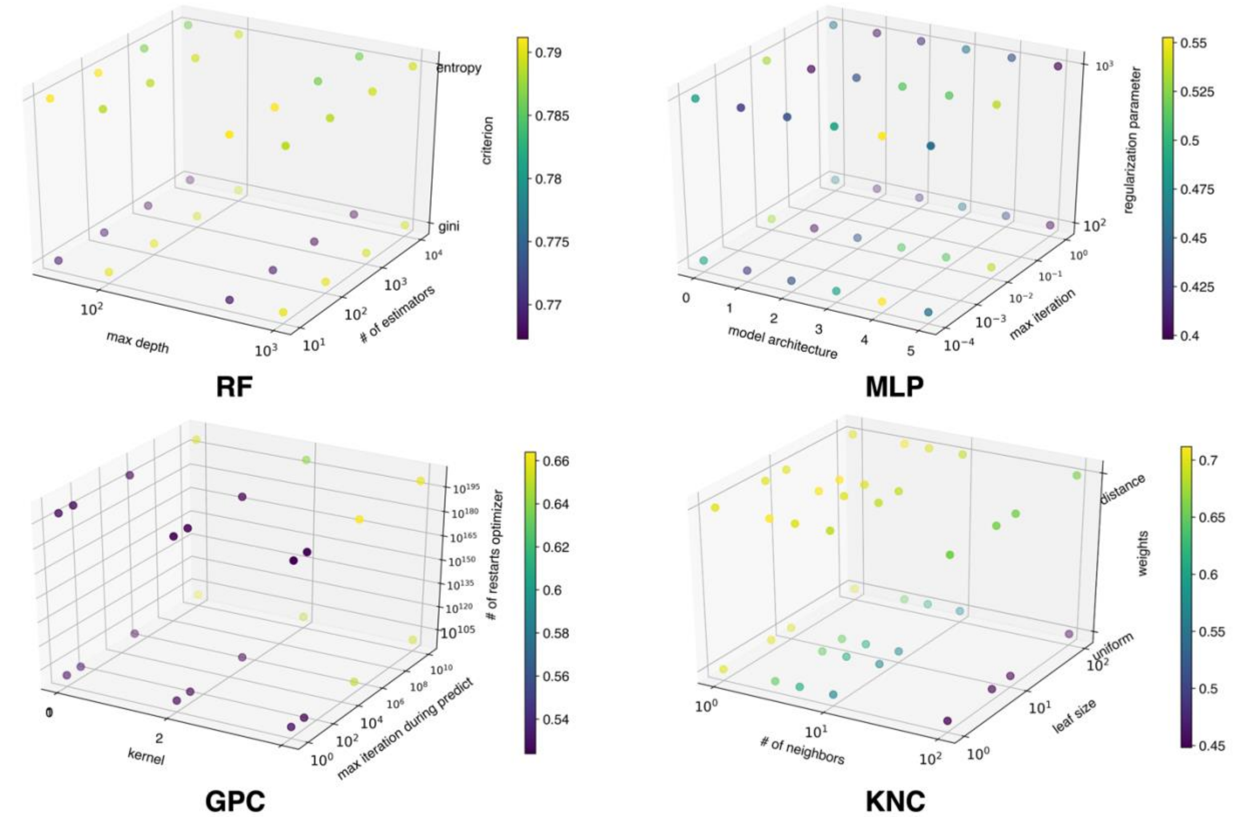

Fig. S1. Hyperparameter grid search for ML algorithms. The hyperparameter combination for optimal RF model is: 'max depth': 50, '\# of estimators': 100, 'criteion': 'gini'; for optimal MLP classifier is: 'regularization parameter': 0.0001, 'model architecture': (128, 256, 128, 16), 'max iteration': 100; for optimal GPC model is: 'kernel': 2RBF(1), 'max iteration during predict': 100, '\# of restarts optimizer': 1; for optimal KNC model is: 'leaf size': 1, '\# of neighbors': 3, 'weights': 'distance'.

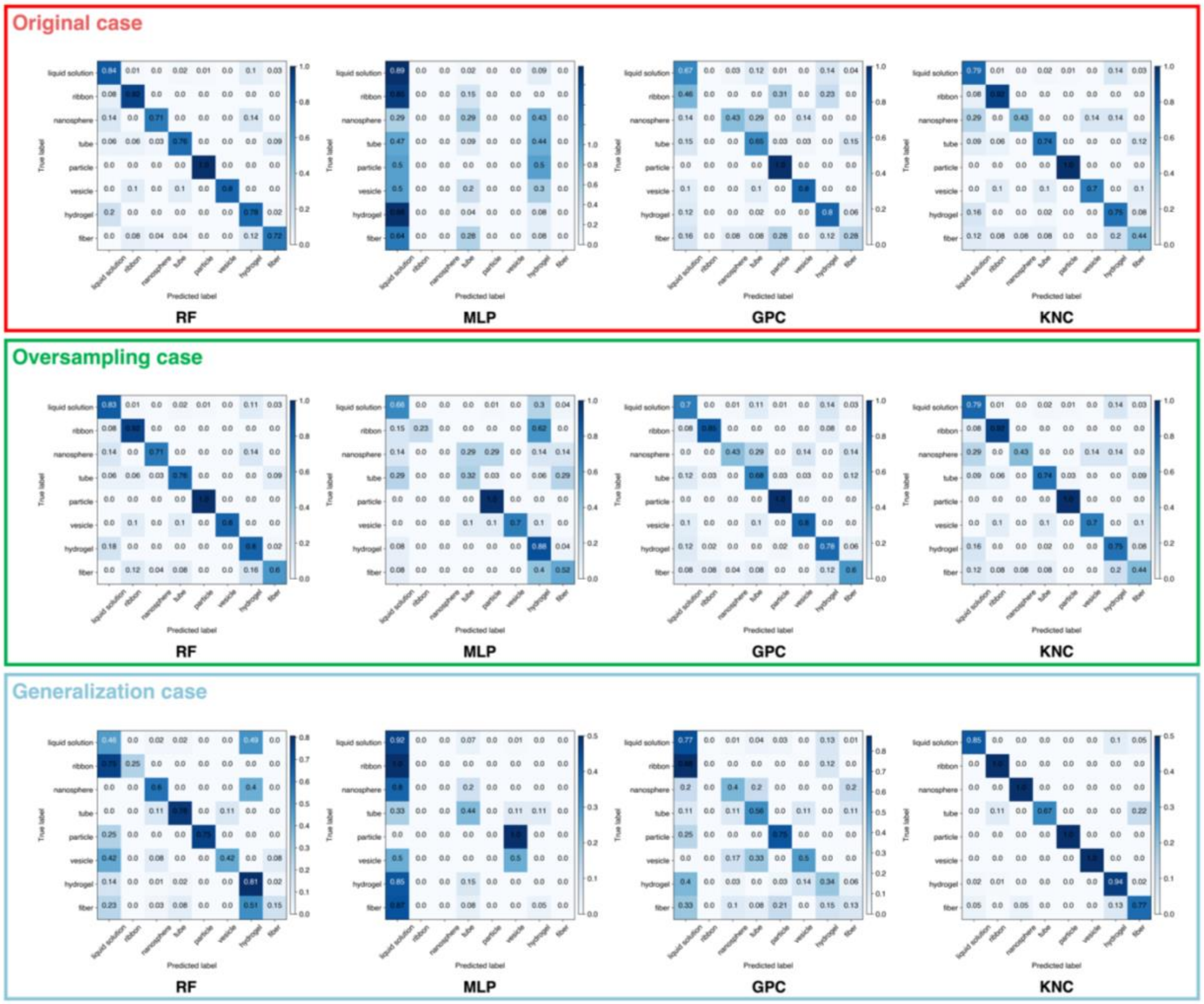

Fig. S2. Confusion matrices. The confusion matrices, which detail the performance outcomes depicted in Fig. 3b, are arranged in a systematic manner: from top to bottom, they are the original case, the oversampling case, and the generalization case. Horizontally, from left to right, the matrices correspond to the RF, MLP, GPC, and KNC models.

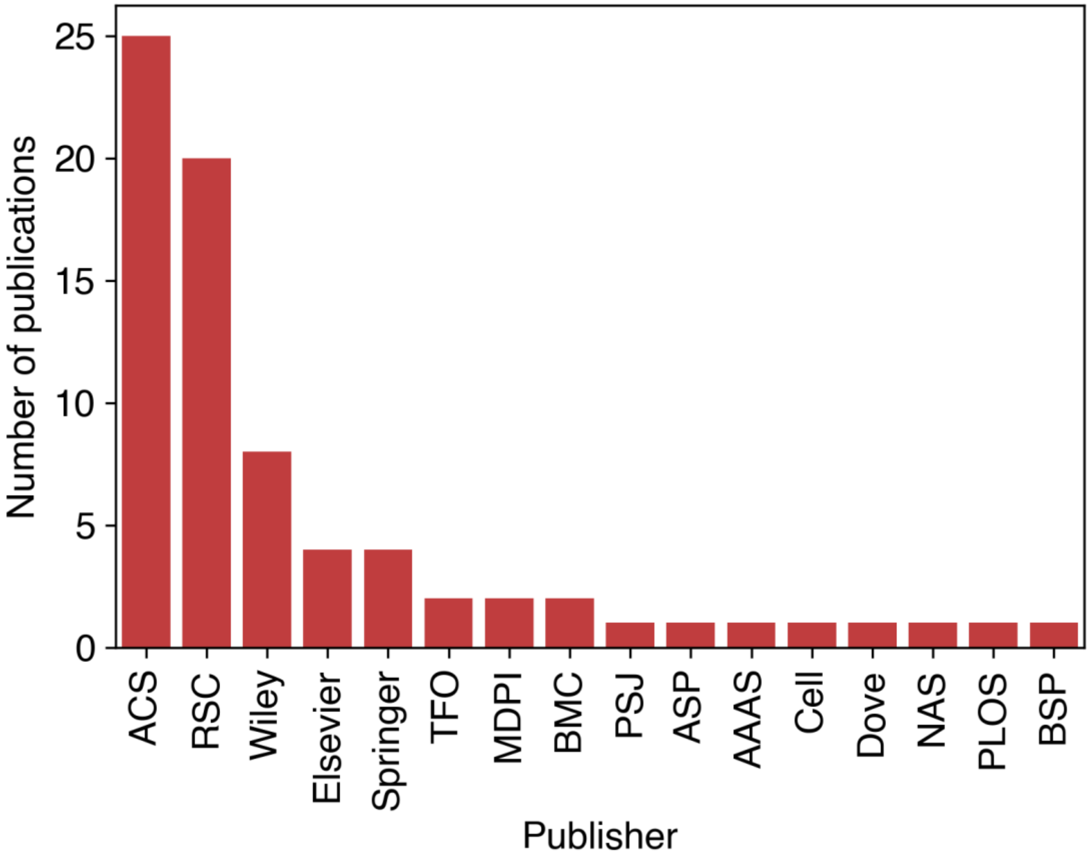

Fig. S3. Publisher information. We here list the number of publications from different journal publishers (see 10.5281/zenodo.14791268). The publishers' abbreviations stand for: "ACS" - "American Chemical Society"; "RSC" - "Royal Society of Chemistry"; "TFO" - Taylor \& Francis Online; "MDPI" - "Multidisciplinary Digital Publishing Institute"; "BMC" - "BioMed Central"; "PSJ" - "Pharmaceutical Society of Japan"; "ASP" - "American Scientific Publisher"; "AAAS" - "American Association for the Advancement of Science"; "NAS" - "National Academy of Sciences"; "BSP" - "Bentham Science Publishers". Based on the histogram, it is noticeable that the publications are mostly from ACS and RSC publishers.

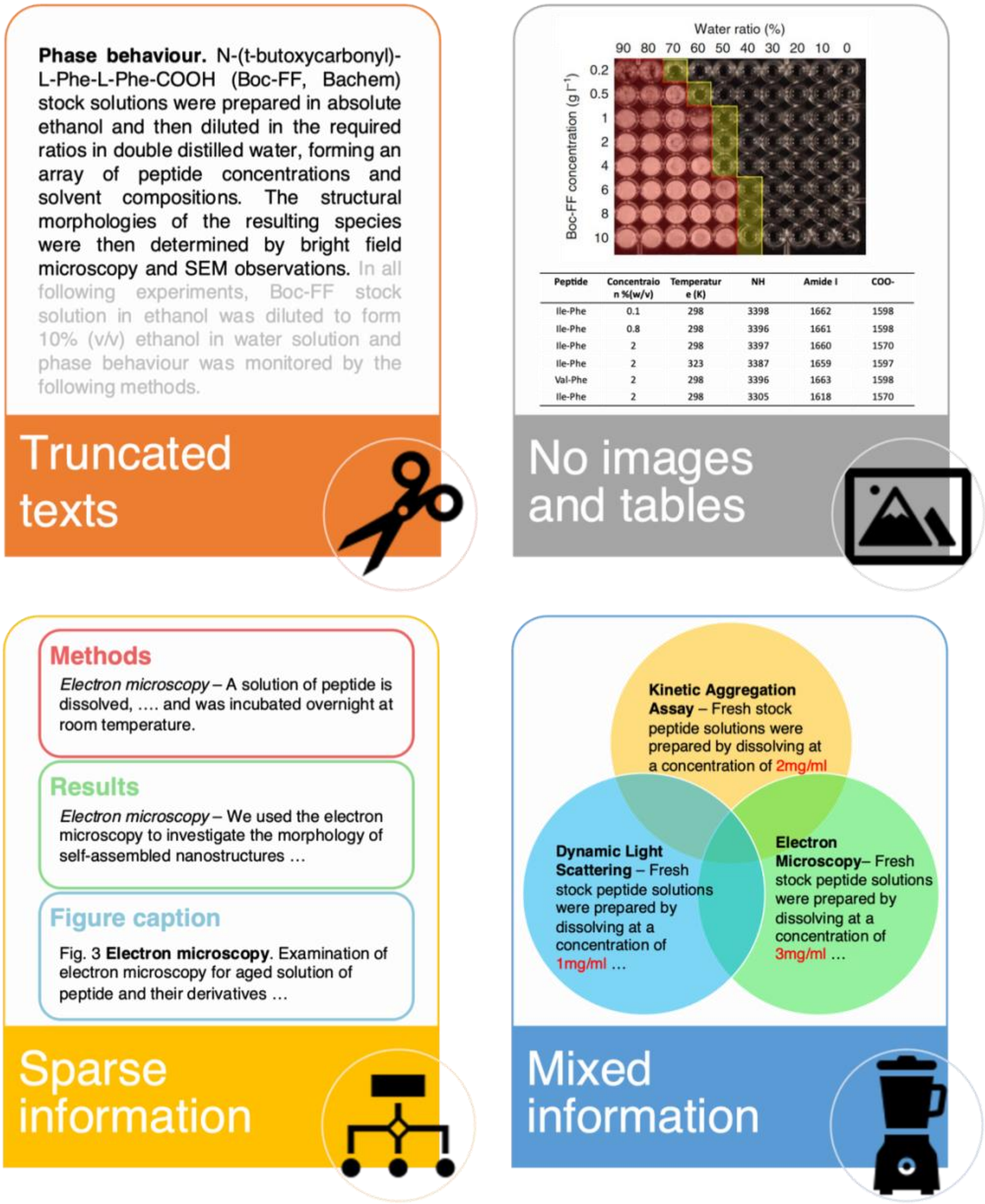

Fig. S4. Limitations of this work. There are 4 main limitations of current work (from left to right): (1) Due to truncated texts, there can be missing information; (2) No tables or images are included which are frequently used to show self-assembly results; (3) Same group of information can be sparse across the whole text which increases the difficulty of text mining; (4) Information is often mixed. For instance, multiple experiments are utilized to investigate the peptide system which results in multiple sets of experimental conditions. The figure in the limitation related to "No images and tables" is reproduced with permission from Ref. *(7)*. The table in the same section is created based on the data from Ref. *(74)*.

Table S1. Data examples. 7 example data entries from two academic publications (PubMed IDs: 23871602 and 18070345) (*68*, *75*).

Data Index	PMID	Peptide sequence	N-terminal modification	C-terminal modification	Non-terminal modification	Category: peptide/conjugate/mixture	Conjugate partner	Thermal process: heating/cooling
1	23871602	FFK	Acetylation	Amidation	None	Peptide	None	No
2	23871602	FYK	Acetylation	Amidation	None	Peptide	None	No
3	23871602	YFK	Acetylation	Amidation	None	Peptide	None	No
4	18070345	FF	Fmoc	None	None	Peptide	None	No
5	18070345	FF	Fmoc	None	None	Peptide	None	No
6	18070345	FF	Fmoc	None	None	Peptide	None	No
7	18070345	FF	Fmoc	None	None	Peptide	None	No

Data Index	PMID	Linear/Cyclic	Solution	Solvent ratio	Concentration	Temperature	PH	Phase
1	23871602	Linear	HFP/H2O	0.05	1	25	5.5	tube
2	23871602	Linear	HFP/H2O	0.05	5	25	5.5	fiber
3	23871602	Linear	HFP/H2O	0.05	5	25	5.5	fiber
4	18070345	Linear	DMSO/PBS	0.05	5.025	37	7.0	None
5	18070345	Linear	DMSO/PBS	0.1	10.05	37	7.0	None
6	18070345	Linear	DMSO/PBS	0.2	5.025	37	7.0	None
7	18070345	Linear	DMSO/PBS	0.4	10.05	37	7.0	None

Table S2. Hyperparameter selection. Hyperparameters for the RF model include: (1) max depth: the maximum depth of the tree; (2) # of estimators: the number of trees in the forest; (3) criterion: the function to measure the quality of a split. For the MLP classifier: (1) model architecture: the sizes of hidden layers as an array; (2) max iteration: maximum number of training iterations; (3) regularization parameters: strength of the L2 regularization term. For the GPC model: (1) kernel: the kernel specifying the covariance function of the Gaussian process; (2) max iteration during predict: the maximum number of iterations in Newton's method for approximating the posterior during predict; (3) # of restarts optimizer: the number of restarts of the optimizer for finding the kernel's parameters which maximize the likelihood. For KNC model: (1) # of neighbors: number of neighbors used for queries; (2) leaf size: leaf size passed to BallTree or KDTree; (3) weights: weight function category.

Model	RF		MLP		GPC		KNC	
	Hyperparameter	Values	Hyperparameter	Values	Hyperparameter	Values	Hyperparameter	Values
#1	max depth	50, 100, 500, 1000	model architecture	(64, 64, 16), (128, 128, 16), (128, 128, 32), (128, 128, 128, 16), (128, 256, 128, 16), (128, 256, 512, 256, 128, 16)	Kernel	RBF(1.0), RBF(2.0), 2RBF(1.0)	# of neighbors	1,3,5,10, 100
#2	# of estimators	10, 100, 1000, 10000	max iteration	100, 1000	max iteration during predict	100, 200	leaf size	1, 5, 10, 100
#3	criterion	"gini", "entropy"	regularization parameter	0.0001, 0.01, 1	# of restart optimizer	0, 1, 5, 10	weights	"uniform", "distance"

Table S3. Key words for paragraph collection. For each paragraph in the text, it is kept if any of the specified keywords are found within it; otherwise, the paragraph is removed.

Relevant entity	Key words
Phase	"gel", "fibr","lamellar", "flower", "fiber", "filament", "spher", "microcrystal", "tube", "rod", "plate",,"flake", "particle", "vesic", "ribbon", "bead", "sheet", "tape", "belt", "film"
PH, Concentration	"dissolve", "dilute", "elute", "wash", "dispers","soluble", "mixed", "added", "pH", "prepare", "concentration","mM","µM", " u00B5M","µL", "u00B5L", "µg","u00B5g", "g/ml", "gyml", "mg/ml", "mgyml", "g/L", "gyL", "kg/L", "kgyL", "w/w", "w/v", "v/v"
Solution	"acetic acid", "acetone", "acetonitrile", "acetyl acetone", "2-aminoethanol", "aniline", "anisole", "benzene", "benzonitrile", "benzyl alcohol", "1-butanol", "2-butanol", "i-butanol", "2-butanone", "t-butyl alcohol", "carbon disulfide", "carbon tetrachloride", "chlorobenzene", "chloroform", "cyclohexane", "cyclohexanol", "cyclohexanone", "di-n-butylphthalate", "1,1-dichloroethane", "1,2-dichloroethane", "diethylamine", "diethylene glycol", "diglyme", "dimethoxyethane", "glyme", "N,N-dimethylaniline", "dimethylformamide", "DMF", "dimethylphthalate", "dimethylsulfoxide", "DMSO", "dioxane", "ethanol", "ether","ethyl acetate", "ethyl acetoacetate", "ethyl benzoate", "ethlyene glycol", "glycerin", "heptane", "1-heptanol", "hexane", "1-hexanol", "methanol", "methyl acetate", "methyl t-butyl ether", "MTBE", "methylene chloride", "1-octanol", "pentane", "1-pentanol", "2-pentanol", "3-pentanol", "2-pentanone", "3-pentanone", "1-propanol", "2-propanol", "pyridine", "tetrahydrofuran", "THF", "toluene", "water", "H2O", "D2O", "o-xylene", "p-xylene", "m-xylene", "heavy water", "water, heavy", "phosphate-buffered saline", "PBS", "sodium phosphate", "hexafluoro-2-propanol", "HFP", "HFIP", "phosphate buffer", "NaOH", "NaCl", "HCl", "glucono delta-lactone", "GdL", "dichloromethane", "1,3-butanediol", "dimethyl carbonate", "pyrimidine", "pyridine", "mesitylene", "octane", "decane", "isopropanol", "glycine".

Table S4. Limitations and solutions. Limitations of current literature mining approach and potential solutions for the future works.

Limitations	Solutions
Given the limit of token numbers, we cut the full texts of academic publications which can eliminate important information from the text.	• The full text can be divided into a series of chunks, allowing LLMs to process them separately rather than all at once.<br>• There are an increasingly number of open-source models with increased token limit as the field evolves.
We only keep the text of publications for data mining. However, figures, tables and videos often contain essential information about self-assembly results	• Multimodal LLMs are capable of simultaneously processing text, images, and videos, offering a comprehensive and integrated approach to literature mining.<br>• Furthermore, given that the graphs within the academic papers often come with captions, contrastive learning can be implemented to fine-tune these pretrained multimodal LLMs using paired text and image data.<br>• Many figures in academic publications are data plots or contain embedded text. To ensure accurate information extraction, supplementary tools such as plot digitization and OCR can be valuable.
The experimental information is often distributed sparsely throughout the full text. For instance, the same group of information, such as observations from electron microscopic examination (see Fig. S4), may be dispersed across various sections of a paper, including Methods, Results, and figure captions.	• During data preprocessing, we can reorganize this relevant information using regular expressions or custom parsing techniques.<br>• In the training stage, the extraction task can be enhanced with clear and task-specific prompts for sparse data collection. (e.g “Extract self-assembly phase from Results section and temperature from Methods section.”)<br>• When performing inference, chain-of-thought (CoT) reasoning can be implemented to extract data in a logical and structured manner (e.g. "Extract experimental methods first and then search for corresponding results").
There is often frequent occurrence of multiple experiments conducted on the same system to explore its morphology, composition, and properties, which confuses the LLMs	• We propose the inclusion of the experimental method as an additional entity for information extraction.<br>• Prompts can be designed to specify the experiments of interest or to instruct the model to distinguish between different experiments.

REFERENCES AND NOTES

1. N. Stephanopoulos, J. H. Ortony, S. I. Stupp, Self-assembly for the synthesis of functional biomaterials. *Acta Mater.* **61**, 912–930 (2013).

2. T. P. J. Knowles, M. J. Buehler, Nanomechanics of functional and pathological amyloid materials. *Nat. Nanotechnol.* **6**, 469–479 (2011).

3. P. J. Horn, C. L. Peterson, Chromatin higher order folding–wrapping up transcription. *Science* **297**, 1824–1827 (2002).

4. T. Aida, E. W. Meijer, S. I. Stupp, Functional supramolecular polymers. *Science* **335**, 813–817 (2012).

5. S. Zhang, Fabrication of novel biomaterials through molecular self-assembly. *Nat. Biotechnol.* **21**, 1171–1178 (2003).

6. J. D. Hartgerink, J. R. Granja, R. A. Milligan, M. R. Ghadiri, Self-assembling peptide nanotubes. *J. Am. Chem. Soc.* **118**, 43–50 (1996).

7. A. Levin, T. O. Mason, L. Adler-Abramovich, A. K. Buell, G. Meisl, C. Galvagnion, Y. Bram, S. A. Stratford, C. M. Dobson, T. P. J. Knowles, E. Gazit, Ostwald's rule of stages governs structural transitions and morphology of dipeptide supramolecular polymers. *Nat. Commun.* **5**, 5219 (2014).

8. K. Matsuura, K. Murasato, N. Kimizuka, Artificial peptide-nanospheres self-assembled from three-way junctions of β-sheet-forming peptides. *J. Am. Chem. Soc.* **127**, 10148–10149 (2005).

9. H. A. Lashuel, S. R. LaBrenz, L. Woo, L. C. Serpell, J. W. Kelly, Protofilaments, filaments, ribbons, and fibrils from peptidomimetic self-assembly: Implications for amyloid fibril formation and materials science. *J. Am. Chem. Soc.* **122**, 5262–5277 (2000).

10. M. J. Sis, M. J. Webber, Drug delivery with designed peptide assemblies. *Trends Pharmacol. Sci.* **40**, 747–762 (2019).

11. S. Koutsopoulos, Self-assembling peptide nanofiber hydrogels in tissue engineering and regenerative medicine: Progress, design guidelines, and applications. *J. Biomed. Mater. Res. A* **104**, 1002–1016 (2016).

12. J. Han, H. Gong, X. Ren, X. Yan, Supramolecular nanozymes based on peptide self-assembly for biomimetic catalysis. *Nano Today* **41**, 101295 (2021).

13. H. A. M. Ardoña, J. D. Tovar, Peptide π-electron conjugates: Organic electronics for biology? *Bioconjug. Chem.* **26**, 2290–2302 (2015).

14. J. Wang, K. Liu, R. Xing, X. Yan, Peptide self-assembly: Thermodynamics and kinetics. *Chem. Soc. Rev.* **45**, 5589–5604 (2016).

15. T. Li, X.-M. Lu, M.-R. Zhang, K. Hu, Z. Li, Peptide-based nanomaterials: Self-assembly, properties and applications. *Bioact. Mater.* **11**, 268–282 (2022).

16. P. W. J. M. Frederix, R. V. Ulijn, N. T. Hunt, T. Tuttle, Virtual screening for dipeptide aggregation: Toward predictive tools for peptide self-assembly. *J. Phys. Chem. Lett.* **2**, 2380–2384 (2011).

17. Z. Li, Y. Zhu, J. B. Matson, pH-responsive self-assembling peptide-based biomaterials: Designs and applications. *ACS Appl. Bio Mater.* **5**, 4635–4651 (2022).

18. M. R. Dreher, A. J. Simnick, K. Fischer, R. J. Smith, A. Patel, M. Schmidt, A. Chilkoti, Temperature triggered self-assembly of polypeptides into multivalent spherical micelles. *J. Am. Chem. Soc.* **130**, 687–694 (2008).

19. A. Nandakumar, Y. Ito, M. Ueda, Solvent effects on the self-assembly of an amphiphilic polypeptide incorporating α-helical hydrophobic blocks. *J. Am. Chem. Soc.* **142**, 20994–21003 (2020).

20. M. J. Krysmann, V. Castelletto, J. E. McKendrick, L. A. Clifton, I. W. Hamley, P. J. F. Harris, S. M. King, Self-assembly of peptide nanotubes in an organic solvent. *Langmuir* **24**, 8158–8162 (2008).

21. T. O. Mason, D. Y. Chirgadze, A. Levin, L. Adler-Abramovich, E. Gazit, T. P. J. Knowles, A. K. Buell, Expanding the solvent chemical space for self-assembly of dipeptide nanostructures. *ACS Nano* **8**, 1243–1253 (2014).

22. C. J. Bowerman, B. L. Nilsson, A reductive trigger for peptide self-assembly and hydrogelation. *J. Am. Chem. Soc.* **132**, 9526–9527 (2010).

23. M. Reches, E. Gazit, Casting metal nanowires within discrete self-assembled peptide nanotubes. *Science* **300**, 625–627 (2003).

24. J. P. Schneider, D. J. Pochan, B. Ozbas, K. Rajagopal, L. Pakstis, J. Kretsinger, Responsive hydrogels from the intramolecular folding and self-assembly of a designed peptide. *J. Am. Chem. Soc.* **124**, 15030–15037 (2002).

25. A. van Teijlingen, T. Tuttle, Beyond tripeptides two-step active machine learning for very large data sets. *J. Chem. Theory Comput.* **17**, 3221–3232 (2021).

26. J. L. Watson, D. Juergens, N. R. Bennett, B. L. Trippe, J. Yim, H. E. Eisenach, W. Ahern, A. J. Borst, R. J. Ragotte, L. F. Milles, B. I. M. Wicky, N. Hanikel, S. J. Pellock, A. Courbet, W. Sheffler, J. Wang, P. Venkatesh, I. Sappington, S. V. Torres, A. Lauko, V. De Bortoli, E. Mathieu, S. Ovchinnikov, R. Barzilay, T. S. Jaakkola, F. D. Maio, M. Baek, D. Baker, De novo design of protein structure and function with RFdiffusion. *Nature* **620**, 1089–1100 (2023).

27. B. Ni, D. L. Kaplan, M. J. Buehler, Generative design of de novo proteins based on secondary-structure constraints using an attention-based diffusion model. *Chem* **9**, 1828–1849 (2023).

28. Z. Yang, Y.-C. Hsu, M. J. Buehler, Generative multiscale analysis of de novo proteome-inspired molecular structures and nanomechanical optimization using a VoxelPerceiver transformer model. *J. Mech. Phys. Solids* **170**, 105098 (2023).

29. W. Lu, D. L. Kaplan, M. J. Buehler, Generative modeling, design, and analysis of spider silk protein sequences for enhanced mechanical properties. *Adv. Funct. Mater.* **34**, 2311324 (2024).

30. A. Ghafarollahi, M. J. Buehler, ProtAgents: Protein discovery *via* large language model multi-agent collaborations combining physics and machine learning. *Digit. Discov.* **3**, 1389–1409 (2024).

31. R. Batra, T. D. Loeffler, H. Chan, S. Srinivasan, H. Cui, I. V. Korendovych, V. Nanda, L. C. Palmer, L. A. Solomon, H. C. Fry, S. K. R. S. Sankaranarayanan, Machine learning overcomes human bias in the discovery of self-assembling peptides. *Nat. Chem.* **14**, 1427–1435 (2022).

32. T. Xu, J. Wang, S. Zhao, D. Chen, H. Zhang, Y. Fang, N. Kong, Z. Zhou, W. Li, H. Wang, Accelerating the prediction and discovery of peptide hydrogels with human-in-the-loop. *Nat. Commun.* **14**, 3880 (2023).

33. S. Kang, M. Kim, J. Sun, M. Lee, K. Min, Prediction of protein aggregation propensity via data-driven approaches. *ACS Biomater Sci. Eng.* **9**, 6451–6463 (2023).

34. J. Wang, Z. Liu, S. Zhao, T. Xu, H. Wang, S. Z. Li, W. Li, Deep learning empowers the discovery of self-assembling peptides with over 10 trillion sequences. *Adv. Sci.* **10**, 2301544 (2023).

35. A.-M. Fernandez-Escamilla, F. Rousseau, J. Schymkowitz, L. Serrano, Prediction of sequence-dependent and mutational effects on the aggregation of peptides and proteins. *Nat. Biotechnol.* **22**, 1302–1306 (2004).

36. R. Zambrano, M. Jamroz, A. Szczasiuk, J. Pujols, S. Kmiecik, S. Ventura, AGGRESCAN3D (A3D): Server for prediction of aggregation properties of protein structures. *Nucleic Acids Res.* **43**, W306–W313 (2015).

37. K. Sankar, S. R. Krystek Jr., S. M. Carl, T. Day, J. K. X. Maier, AggScore: Prediction of aggregation-prone regions in proteins based on the distribution of surface patches. *Proteins* **86**, 1147–1156 (2018).

38. W. X. Zhao, K. Zhou, J. Li, T. Tang, X. Wang, Y. Hou, Y. Min, B. Zhang, J. Zhang, Z. Dong, Y. Du, C. Yang, Y. Chen, Z. Chen, J. Jiang, R. Ren, Y. Li, X. Tang, Z. Liu, P. Liu, J.-Y. Nie, J.-R. Wen, Survey of Large Language Models. arXiv:2303.18223 [cs.CL] (2023).

39. A. Vaswani, N. Shazeer, N. Parmar, J. Uszkoreit, L. Jones, A. N. Gomez, Ł. Kaiser, I. Polosukhin, Attention is all you need, in *Proceedings of the 31st International Conference on Neural Information Processing Systems* (Curran Associates Inc., 2017), pp. 6000–6010.

40. M. J. Buehler, Accelerating Scientific Discovery with Generative Knowledge Extraction, Graph-Based Representation, and Multimodal Intelligent Graph Reasoning. arXiv:2403.119961 [cs.LG] (2024).

41. T. B. Brown, B. Mann, N. Ryder, M. Subbiah, J. Kaplan, P. Dhariwal, A. Neelakantan, P. Shyam, G. Sastry, A. Askell, S. Agarwal, A. Herbert-Voss, G. Krueger, T. Henighan, R. Child, A. Ramesh, D. M. Ziegler, J. Wu, C. Winter, C. Hesse, M. Chen, E. Sigler, M. Litwin, S. Gray, B. Chess, J. Clark, C. Berner, S. McCandlish, A. Radford, I. Sutskever, D. Amodei, Language Models are Few-Shot Learners. arXiv:2005.14165 [cs.CL] (2020).

42. H. Touvron, L. Martin, K. Stone, P. Albert, A. Almahairi, Y. Babaei, N. Bashlykov, S. Batra, P. Bhargava, S. Bhosale, D. Bikel, L. Blecher, C. C. Ferrer, M. Chen, G. Cucurull, D. Esiobu, J. Fernandes, J. Fu, W. Fu, B. Fuller, C. Gao, V. Goswami, N. Goyal, A. Hartshorn, S. Hosseini, R. Hou, H. Inan, M. Kardas, V. Kerkez, M. Khabsa, I. Kloumann, A. Korenev, P. S. Koura, M.-A. Lachaux, T. Lavril, J. Lee, D. Liskovich, Y. Lu, Y. Mao, X. Martinet, T. Mihaylov, P. Mishra, I. Molybog, Y. Nie, A. Poulton, J. Reizenstein, R. Rungta, K. Saladi, A. Schelten, R. Silva, E. M. Smith, R. Subramanian, X. E. Tan, B. Tang, R. Taylor, A. Williams, J. X. Kuan, P. Xu, Z. Yan, I. Zarov, Y. Zhang, A. Fan, M. Kambadur, S. Narang, A. Rodriguez, R. Stojnic, S. Edunov, T. Scialom, Llama 2: Open Foundation and Fine-Tuned Chat Models. arXiv:2307.09288 [cs.CL] (2023).

43. V. Tshitoyan, J. Dagdelen, L. Weston, A. Dunn, Z. Rong, O. Kononova, K. A. Persson, G. Ceder, A. Jain, Unsupervised word embeddings capture latent knowledge from materials science literature. *Nature* **571**, 95–98 (2019).

44. T. Gupta, M. Zaki, N. M. A. Krishnan, Mausam, MatSciBERT: A materials domain language model for text mining and information extraction. *NPJ Comput. Mater.* **8**, 102 (2022).

45. L. Foppiano, G. Lambard, T. Amagasa, M. Ishii, Mining experimental data from Materials Science literature with Large Language Models: an evaluation study. arXiv:2401.11052 [cs. CL] (2024).

46. R. K. Luu, M. J. Buehler, BioinspiredLLM: Conversational large language model for the mechanics of biological and bio-inspired materials. *Adv. Sci.* **11**, 2306724 (2024).

47. M. J. Buehler, MechGPT, a language-based strategy for mechanics and materials modeling that connects knowledge across scales, disciplines, and modalities. *Appl. Mech. Rev.* **76**, 21001 (2024).

48. M. J. Buehler, Cephalo: Multi-Modal Vision-Language Models for Bio-Inspired Materials Analysis and Design. arXiv:2405.19076 [cs.CV] (2024).

49. D. Mathur, H. Kaur, A. Dhall, N. Sharma, G. P. S. Raghava, SAPdb: A database of short peptides and the corresponding nanostructures formed by self-assembly. *Comput. Biol. Med.* **133**, 104391 (2021).

50. P. Tamamis, L. Adler-Abramovich, M. Reches, K. Marshall, P. Sikorski, L. Serpell, E. Gazit, G. Archontis, Self-assembly of phenylalanine oligopeptides: insights from experiments and simulations. *Biophys. J.* **96**, 5020–5029 (2009).

51. N. V. Chawla, K. W. Bowyer, L. O. Hall, W. P. Kegelmeyer, SMOTE: Synthetic minority over-sampling technique. *J. Artif. Int. Res.* **16**, 321–357 (2002).

52. S. M. Lundberg, S.-I. Lee, A Unified Approach to Interpreting Model Predictions, in *Advances in Neural Information Processing Systems 30*, I. Guyon, U. V. Luxburg, S. Bengio,

H. Wallach, R. Fergus, S. Vishwanathan, R. Garnett, Eds. (Curran Associates Inc., 2017), pp. 4765–4774.

53. pdfminer developers, pdfminer.six PDF parser and analyzer (2024).

54. OpenAI, GPT-3.5-turbo-0125 (2023).

55. S. Marchesan, Y. Qu, L. J. Waddington, C. D. Easton, V. Glattauer, T. J. Lithgow, K. M. Mc Lean, J. S. Forsythe, P. G. Hartley, Self-assembly of ciprofloxacin and a tripeptide into an antimicrobial nanostructured hydrogel. *Biomaterials* **34**, 3678–3687 (2013).

56. C. Fare, P. Fenner, M. Benatan, A. Varsi, E. O. Pyzer-Knapp, A multi-fidelity machine learning approach to high throughput materials screening. *NPJ Comput. Mater.* **8**, 257 (2022).

57. P. W. J. M. Frederix, G. G. Scott, Y. M. Abul-Haija, D. Kalafatovic, C. G. Pappas, N. Javid, N. T. Hunt, R. V. Ulijn, T. Tuttle, Exploring the sequence space for (tri-)peptide self-assembly to design and discover new hydrogels. *Nat. Chem.* **7**, 30–37 (2015).

58. B. Sanchez-Lengeling, A. Aspuru-Guzik, Inverse molecular design using machine learning: Generative models for matter engineering. *Science* **361**, 360–365 (2018).

59. Y.-C. Hsu, Z. Yang, M. J. Buehler, Generative design, manufacturing, and molecular modeling of 3D architected materials based on natural language input. *APL Mater.* **10**, 41107 (2022).

60. R. Xing, S. Li, N. Zhang, G. Shen, H. Möhwald, X. Yan, Self-assembled injectable peptide hydrogels capable of triggering antitumor immune response. *Biomacromolecules* **18**, 3514–3523 (2017).

61. C. Guo, Y. Luo, R. Zhou, G. Wei, Triphenylalanine peptides self-assemble into nanospheres and nanorods that are different from the nanovesicles and nanotubes formed by diphenylalanine peptides. *Nanoscale* **6**, 2800–2811 (2014).

62. I. Bhardwaj, D. Jha, P. Admane, A. K. Panda, V. Haridas, Self-assembling tryptophan-based designer peptides as intracellular delivery vehicles. *Bioorg. Med. Chem. Lett.* **26**, 672–676 (2016).

63. C. G. Pappas, P. W. J. M. Frederix, T. Mutasa, S. Fleming, Y. M. Abul-Haija, S. M. Kelly, A. Gachagan, D. Kalafatovic, J. Trevino, R. V. Ulijn, S. Bai, Alignment of nanostructured tripeptide gels by directional ultrasonication. *Chem. Commun.* **51**, 8465–8468 (2015).

64. R. P. Lyon, W. M. Atkins, Self-assembly and gelation of oxidized glutathione in organic solvents. *J. Am. Chem. Soc.* **123**, 4408–4413 (2001).

65. G. Baskar, M. Ravi, J. J. Panda, A. Khatri, B. Dev, R. Santosham, S. Sathiya, C. S. Babu, V. S. Chauhan, S. K. Rayala, G. Venkatraman, Efficacy of dipeptide-coated magnetic nanoparticles in lung cancer models under pulsed electromagnetic field. *Cancer Invest.* **35**, 431–442 (2017).

66. L. Adler-Abramovich, E. Gazit, Controlled patterning of peptide nanotubes and nanospheres using inkjet printing technology. *J. Pept. Sci.* **14**, 217–223 (2008).

67. C. A. E. Hauser, R. Deng, A. Mishra, Y. Loo, U. Khoe, F. Zhuang, D. W. Cheong, A. Accardo, M. B. Sullivan, C. Riekel, J. Y. Ying, U. A. Hauser, Natural tri- to hexapeptides self-assemble in water to amyloid β-type fiber aggregates by unexpected α-helical intermediate structures. *Proc. Natl. Acad. Sci. U.S.A.* **108**, 1361–1366 (2011).

68. T. Liebmann, S. Rydholm, V. Akpe, H. Brismar, Self-assembling Fmoc dipeptide hydrogel for in situ 3D cell culturing. *BMC Biotechnol.* **7**, 88 (2007).

69. S. Yadav, V. Rai, M. Mahato, M. Singh, R. S. Deka, K. A. Sharma, Vitamin E–TPGS stabilized self-assembled tripeptide nanostructures for drug delivery. *Curr. Top. Med. Chem.* **15**, 1227–1235 (2015).

70. F. Pedregosa, G. Varoquaux, A. Gramfort, B. Michel, V. Thirion, O. Grisel, M. Blondel, R. Prettenhofer, P. Weiss, V. Dubourg, J. Vanderplas, A. Passos, D. Cournapeau, M. Brucher, M.

Perrot, E. Duchesnay, Scikit-learn: Machine learning in Python. *J. Mach. Learn. Res.* **12**, 2825–2830 (2011).

71. G. Lemaître, F. Nogueira, C. K. Aridas, Imbalanced-learn: A python toolbox to tackle the curse of imbalanced datasets in machine learning. *J. Mach. Learn. Res.* **18**, 1–5 (2017).

72. S. M. Lundberg, G. Erion, H. Chen, A. DeGrave, J. M. Prutkin, B. Nair, R. Katz, J. Himmelfarb, N. Bansal, S.-I. Lee, From local explanations to global understanding with explainable AI for trees. *Nat. Mach. Intell.* **2**, 2522–5839 (2020).

73. E. J. Hu, Y. Shen, P. Wallis, Z. Allen-Zhu, Y. Li, S. Wang, L. Wang, W. Chen, LoRA: Low-Rank Adaptation of Large Language Models, in *International Conference on Learning Representations (ICLR)* (ICLR, 2022).

74. N. S. de Groot, Parella, T. Parella, F. X. Aviles, S. Ventura, Ile-Phe dipeptide self-assembly: Clues to amyloid formation. *Biophys. J.* **92**, 1732–1741 (2007).

75. M. Cao, C. Cao, L. Zhang, D. Xia, H. Xu, Tuning of peptide assembly through force balance adjustment. *J. Colloid Interface Sci.* **407**, 287–295 (2013).
